# OpenEMMU: A versatile, open-source EdU multiplexing methodology for studying DNA replication and cell cycle dynamics

**DOI:** 10.1016/j.isci.2025.113380

**Published:** 2025-08-16

**Authors:** Osvaldo Contreras, Chris Thekkedam, John Zaunders, Ismael Aguirre-MacLennan, Nicholas J. Murray, Anai Gonzalez-Cordero, Richard P. Harvey

**Affiliations:** 1 Victor Chang Cardiac Research Institute, Darlinghurst, NSW 2010, Australia; 2 School of Clinical Medicine, Faculty of Medicine & Health, UNSW Sydney, Kensington, NSW 2052, Australia; 3 Centre for Applied Medical Research, St Vincent’s Hospital, Sydney, NSW, Australia; 4 Stem Cell Medicine and Stem Cell and Organoid Facility, Children’s Medical Research Institute, Faculty of Medicine and Health, The University of Sydney, Westmead, NSW 2145, Australia; 5 School of Biotechnology and Biomolecular Science, Faculty of Science, UNSW Sydney, Kensington, NSW 2052, Australia

**Keywords:** Biochemistry, Cell biology, Developmental biology, Computational bioinformatics

## Abstract

5-Ethynyl-2′-deoxyuridine (EdU) has revolutionized DNA replication and cell cycle analyses through fast, efficient click chemistry detection. However, commercial EdU kits suffer from high costs, proprietary formulations, limited antibody multiplexing capabilities, and difficulties with larger biological specimens. Here, we present OpenEMMU (Open-source EdU Multiplexing Methodology for Understanding DNA replication dynamics), an optimized, affordable, and user-friendly click chemistry platform utilizing off-the-shelf reagents. OpenEMMU enhances efficiency, brightness, and multiplexing capabilities of EdU staining with both non-conjugated and conjugated antibodies across diverse cell types, including T cell activation and proliferation assays. We validated its effectiveness for the fluorescent imaging of nascent DNA synthesis in developing embryos and organs, including embryonic heart, forelimbs, and 3D hiPSC-derived cardiac organoids. OpenEMMU also enabled the deep-tissue 3D imaging of DNA synthesis in zebrafish larvae and under replication stress in embryos at high spatial resolution. This approach opens new avenues for understanding organismal development, cell proliferation, and DNA replication dynamics with unprecedented precision and flexibility.

## Highlights

• An affordable, open-source toolkit for studying DNA replication and cell cycle profiling
• Optimized click chemistry enhances EdU signal and antibody multiplexing with IBEX
• Profiles human T cell activation and proliferation in response to antigens and infections
• Enables the 3D imaging of DNA replication in whole organs, cardiac organoids, and zebrafish

## Graphical abstract

**Figure:**
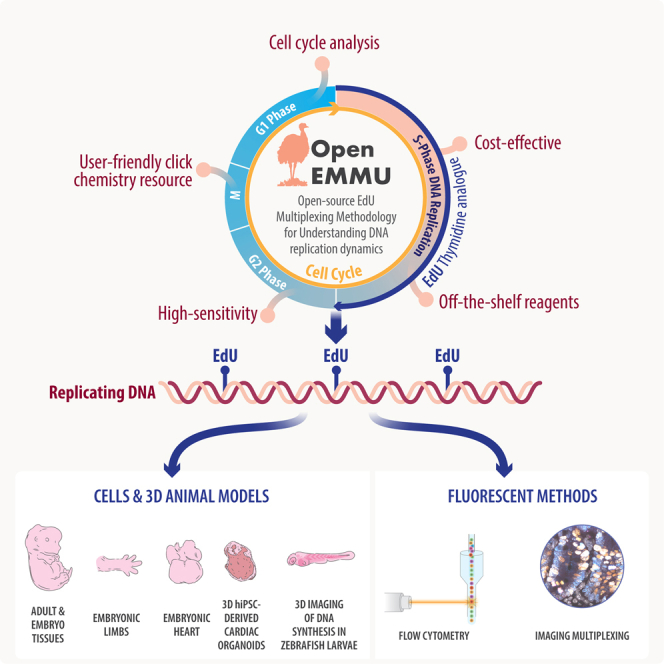

## Introduction

The cell cycle is a fundamental process in cell division, crucial for cell commitment, tissue morphogenesis, development, and homeostasis. Understanding its core phases–G1, S, G2, and Mitosis–and their regulatory mechanisms is essential for explaining congenital malformations and adult diseases, including cancer.^1^ Researchers use various tools and strategies to map the progression and phases of the cell cycle, including the immunodetection of Ki67 for cell cycle competence,^2^ phosphorylated-Histone H3 for mitotic cells,^3^ Aurora B for cytokinesis,^4^ nucleic acid dyes for DNA content analysis^5^ and FUCCI reporters for cell cycle dynamics.^6^

DNA replication occurs during the S phase of the cell cycle, ensuring the accurate duplication of genetic information.^7^ To study DNA replication, researchers have traditionally used thymidine analogs that are incorporated into newly synthesized DNA.^8^ These analogs have provided insights into DNA replication rate, extent, and genome location, with thousands of studies demonstrating their use. Early methods used radioactive thymidine incorporation with autoradiographic detection,^9^ whereas later, the incorporation of BrdU, a halogenated thymidine analog, was detected with antibodies^10^^,^^11^^,^^12^. More recently, EdU has gained popularity for its simpler, faster, and more sensitive detection using click chemistry.^9^^,^^13^ Unlike BrdU detection, which uses harsh DNA denaturation conditions necessary for antigen retrieval, EdU click-chemistry-based detection minimizes cellular damage and other artifacts, is more efficient than traditional antibody-based approaches, and is well-suited for high-throughput studies, making EdU a preferred choice for DNA replication research.^9^^,^^14^^,^^15^

EdU detection utilizes click chemistry, a groundbreaking innovation that enables fast and efficient covalent conjugation of molecular entities.^13^ This technique was recognized with the 2022 Nobel Prize in Chemistry.^16^^,^^17^^,^^18^ Specifically, the Cu(I)-Catalyzed Azide−Alkyne Cycloaddition (CuAAC) reaction demonstrates high catalytic efficacy when combined with reductants, proceeds rapidly and reliably under mild, often aqueous conditions^,^^17^ and achieves near-quantitative yields in forming stable triazole products,^19^ making it ideal for biological systems. The specificity of the reaction is enhanced by the bioorthogonal azide and alkyne groups, and the stability of the triazole linkage, which is resistant to common chemical degradations, makes it suitable for long-term applications such as drug delivery and extended biomolecular labeling. Its versatility extends beyond biology, encompassing polymer science, drug and pharmaceutical development, and materials chemistry.^13^^,^^20^

Since its introduction in 2008,^21^ EdU has become a widely adopted tool for studying DNA replication, cell proliferation, and differentiation. When combined with DNA content assessment, it is particularly effective for identifying different cell cycle phases.^22^ Notable examples of its application include studies on the proliferative nature of the stem cell gut niche,^21^^,^^23^^,^^24^ dividing stem cells,^15^ and adult skeletal muscle stem cell-mediated regeneration and quiescence.^25^^,^^26^ Additionally, EdU has been instrumental in research on embryonic and adult neurogenesis,^27^^,^^28^^,^^29^ the proliferative response of adult cardiac fibroblasts to myocardial damage,^30^ and root plant growth.^31^^,^^32^ EdU has also significantly contributed to cancer research and has been used to evaluate mammalian cardiomyocyte DNA synthesis and chromosome polyploidization after birth.^33^ Furthermore, it has been applied in super-resolution microscopy of DNA replication sites^34^^,^^35^ and chromosome labeling.^36^ Beyond standard labeling, EdU enables methods such as iPOND (isolation of proteins on nascent DNA) to perform spatiotemporal analysis of proteins at replication forks or on chromatin in cultured cells.^37^

Recognizing its potential, several biotechnology companies have developed commercial EdU kits (e.g., Invitrogen by Life Technologies) for imaging and flow cytometry. However, EdU kits have several limitations that hinder scientific progress, including proprietary reagents and their concentrations, and high cost (USD 700-1,200 for 50 reactions/tubes). The need for separate kits for imaging and flow cytometry further limits flexibility and adds additional expenses when employing both techniques. Pipelines are not straightforward when using antibodies for parallel marker immunodetection, whether primary or conjugated, or other staining procedures. EdU kits expire, and most are limited to detecting 4–5 colors, restricting their multiplexing capabilities. In addition, methods are not optimized for use in whole organs or tissues in 3D, limiting analysis of larger and more complex biological structures or whole organisms. Overall, these limitations underscore the need for more affordable, open-source, and versatile alternatives, particularly for applications involving complex biological structures.

To address these limitations, we have developed **Open**-source **E**dU **M**ultiplexing **M**ethodology for **U**nderstanding DNA replication dynamics (OpenEMMU), a flexible and user-friendly clickable method, designed for harmonizing high-parameter flow cytometry and various cutting-edge imaging modalities, utilizing commonly available and inexpensive reagents. We established OpenEMMU for the fluorescent staining of clickable EdU in 8 different cell types, the adult gut stem cell niche, splenocytes, bone marrow cells, activated human T cells, whole developing organs including the embryonic heart and forelimb, and 3D self-assembled human organoids. Moreover, OpenEMMU was compatible with multiplexed imaging using “iterative bleaching extends multiplexity” or IBEX^38^ and enabled high-resolution 3D evaluation of nascent DNA synthesis in whole zebrafish larvae, which was not possible with traditional click staining methods. OpenEMMU allows for in-house optimization and validation at an unprecedented low cost, making it superior to commercially available EdU kits for DNA replication studies in simple and complex biological systems.

## Results

### Optimizing OpenEMMU for S-phase DNA replication profiling using Cu(I)-catalyzed azide−alkyne cycloaddition click chemistry

**Figure 1:**
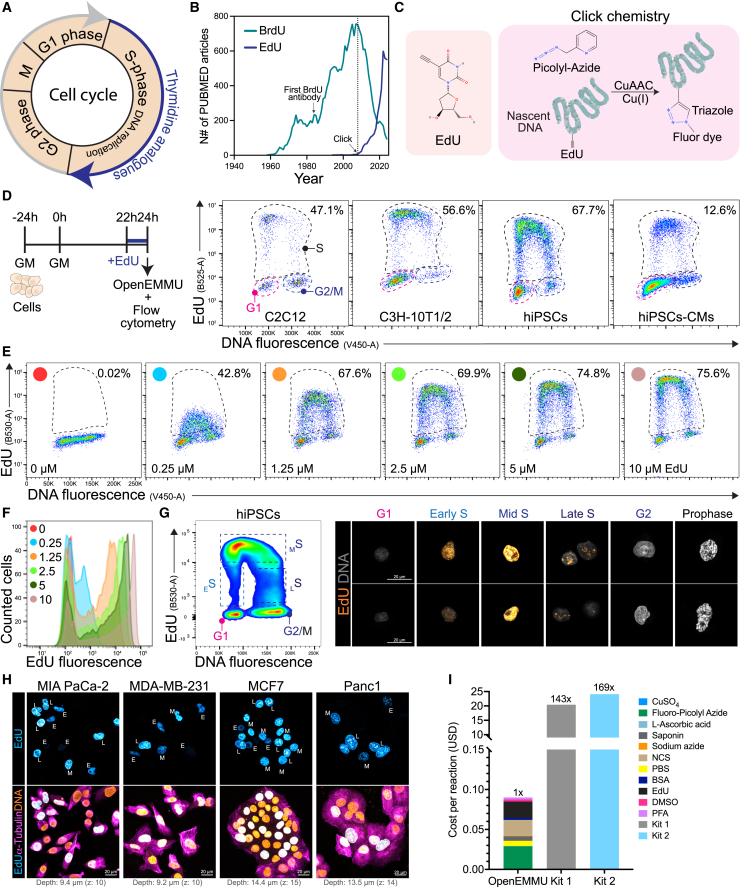
Application of OpenEMMU to normal and cancer cell lines for analysis of DNA replication (A) Diagram outlining the cell cycle phases, highlighting the S phase where thymidine analogs are incorporated during DNA replication. (B) Graph depicting the annual number of PUBMED articles mentioning BrdU or EdU. (C) Illustration of the EdU molecule and CuAAC reaction in cells that have incorporated EdU into nascent DNA. (D) Flow cytometry results show the proportion of EdU-labeled cells after a 2-h pulse in indicated cell lines (*n* = 3). (E) Relationship between EdU fluorescence intensity and the proportion of EdU+ cells at varying EdU concentrations in hiPSCs (*n* = 3). (F) Comparison of EdU and DNA fluorescence intensity at different EdU concentrations from (E). (G) Flow cytometry analysis of hiPSCs following a 1-h EdU pulse. The dotted lines indicate the gating strategy employed for both FACS and subsequent confocal microscopy of sorted cells (right panels) (*n* = 3). (H) Confocal microscopy images of indicated EdU+ cancer cell lines after a 2-h EdU pulse, with α-Tubulin immunolabeling and DNA staining (*n* = 3). E: Early DNA replication (S-phase); M: Mid DNA replication; L: Late DNA replication. (I) Cost per 0.5 mL reaction comparing OpenEMMU against two commercially available kit options (Invitrogen, kit 1; Vector Labs, kit 2).

Since its discovery and application to the study of DNA synthesis and cell cycle dynamics (Figure 1A),^21^ EdU usage has steadily increased, while BrdU usage has substantially declined (Figure 1B). As EdU detection relies on click chemistry, we aimed to develop an enhanced, open-source, and cost-effective CuAAC-based EdU click reaction requiring common laboratory reagents and basic laboratory skills (Figure 1C).

We conducted a systematic evaluation of critical reagents and reaction conditions to refine CuAAC click chemistry for the precise and efficient detection of nuclear DNA synthesis in proliferating cells. This analysis assessed their effects on labeling efficiency and signal quality across diverse cell lines and species (Figures 1 and S1). To improve usability, we created detailed step-by-step guides for flow cytometry and imaging (STAR Methods; Figures S2 and S3A), incorporating antibody immunolabeling and immunohistochemistry. In our flow cytometry and imaging studies, cells were treated with EdU for 2h at increasing concentrations (0-10 μM) and after fixation in PFA were incubated with an optimized OpenEMMU click reaction mixture, consisting of Cu-chelating azide dye (AZDye488/555/633/680 Picolyl Azide; 0.2 μM), copper catalyst (CuSO_4_.5H2O; 0.8 mM), and reducing agent (L-ascorbic acid; 1 mg/mL) in PBS for 30 min (STAR Methods; Figures 1D and 1E). Our evaluation showed that CuSO_4_ was a limiting reagent, and DNA replicating cells were not labeled properly below 800 μM (Figures 1E, 1F, and S1A–S1C). Whereas 400 μM produced a significant number of EdU-labeled cells, labeling efficiency was reduced compared to 800 μM in our standardized conditions (Figure S1C). Increasing the CuSO_4_ concentration to 2 mM did not improve labeling but diminished DNA content fluorescence intensity as measured by Vybrant Violet DNA dye fluorescence (Figure S1C).

To further optimize the CuAAC-based click reaction, we quantified the click reaction efficiency using L-ascorbic acid as the reducing agent. Among the tested concentrations, 0.5 mg/mL and higher were effective, leading us to select 1 mg/mL as our working concentration (Figure S1D). 0.1 mg/mL did not facilitate the CuAAC click reaction. These findings highlight the importance of reducing agent concentration for efficient click chemistry.^19^ We also investigated the efficiency of the copper-chelating organic picolyl azide in the CuAAC click reaction. Picolyl azides were effective at concentrations between 0.1 and 0.5 μM but caused overstaining of non-EdU cells at higher concentrations (2–10 μM) (Figure S1E). Thus, increasing the concentration of AZDye-conjugated picolyl azide did not improve staining or separation but reduced the signal-to-noise ratio. Therefore, we selected 0.2 μM as the optimal working concentration for picolyl azides (tested AZDye 488/555/633/680).

We then compared two bovine serum types in our adapted permeabilization and wash buffer to further refine our protocol. Both Newborn Calf Serum (NCS) at 2% and Fetal Bovine Serum (FBS) at 4% effectively supported EdU labeling (Figure S1F). NCS was selected due to its lower cost compared to FBS, although either can be used depending on the researchers’ needs. We also explored various DNA dyes and observed that OpenEMMU was compatible with all tested. However, Vybrant DyeCycle Violet was significantly brighter than Hoechst 33342, Hoechst 33258, and DAPI, even at lower concentrations (5 μM Vybrant DyeCycle Violet versus 18 μM for the other DNA dyes) (Figure S1G).

### OpenEMMU enables the cost-effective investigation of DNA replication in multiple cell types using flow cytometry and fluorescence microscopy

Two hours of EdU treatment at 10 μM effectively labels various cell types, including C2C12 myoblasts, C3H10T1/2 mesenchymal stromal cells, human-induced pluripotent stem cells (hiPSCs), and hiPSC-derived ventricular cardiomyocytes at day 12 of differentiation (Figures 1D and S3B). These cell types exhibit varying degrees of DNA replication rate and S-phase cell proportions (approximately 47, 57, 68, and ∼13% of total cells**,** respectively). The horseshoe-shaped EdU/DNA bivariate distribution allows for the identification of cells in the G1 phase (2n DNA, no EdU), S phase (DNA replicating, EdU^+^), and the G2/M phases (4n DNA, no EdU) of the cell cycle by combining EdU incorporation and DNA fluorescence detection using Vybrant Violet (Figure 1D). We confirmed that the optimal EdU concentration range of 5–10 μM is necessary for accurately labeling DNA-replicating hiPSCs, as lower concentrations (below 5 μM) significantly reduce EdU fluorescence intensity and the proportion of S-phase cells detected (Figures 1E and 1F).

To investigate the kinetics of EdU labeling and the influence of pulse duration, we performed time-course EdU pulse experiments in undifferentiated hiPSCs analyzed by quantitative flow cytometry. The percentage of EdU-positive cells reached a plateau after approximately 20–30 min of incubation (Figures S4A and S4B), indicating that pulse lengths within this range efficiently label the majority of cells actively synthesizing DNA. In contrast, the mean fluorescence intensity (MFI) of the EdU signal within labeled cells continued to increase significantly with longer pulse durations (Figures S4C and S4D). This observation demonstrates that although a relatively short pulse is sufficient to identify all S-phase cells, prolonged incubation allows for greater EdU incorporation and a stronger signal, likely due to the continued uptake and utilization of the intracellular EdU pool.^39^ Therefore, our unified clickable method delivers excellent results for both flow cytometry and fluorescence imaging, overcoming the limitations of most commercial EdU kits, which typically support only one of these techniques.

OpenEMMU also enabled fluorescence-activated cell sorting (FACS) of distinct DNA replicating cells, specifically early, mid, and late S-phase cells, using the horseshoe-shaped EdU/DNA bivariate fluorescence as a proxy (Figure 1G). FACS-assisted separation followed by cytospin of EdU^+^ hiPSCs (1h EdU) allowed us to distinguish distinct DNA replicating hiPSC populations and identify specific regions of DNA synthesis within the nuclear DNA. Notably, the intensity of EdU fluorescence and DNA content allowed the differentiation of compartmentalized DNA replication dynamics among early, mid, and late replicating cells (Figure 1G). Open, less condensed euchromatic DNA replicates first, followed by more condensed heterochromatic DNA, which typically replicates during the late S-phase.^40^^,^^41^ Early replicating DNA in hiPSCs undergoing the S-phase exhibited a less intense, dispersed, and punctate DNA synthesis pattern largely excluding the nucleolus, whereas mid-replicating cells displayed larger and more abundant DNA replication foci concentrated at the nuclear periphery and within the nucleoplasm. In contrast, late S-phase cells showed a patchier and more segregated DNA replication pattern, particularly within the nucleolus and other late-replicating regions and chromosomes (Figure 1G). Co-staining with NUCLEOLIN (for nucleoli) and EdU in hiPSCs, using a brief 10-min labeling period, enabled the precise identification of replicative events at different stages of the S-phase (Figure S5A). Thus, OpenEMMU enables S-phase cell profiling by providing high-resolution discrimination and evaluation of DNA synthesis’s spatial domains.

To explore other relevant cell types, we examined various cancer cell lines (Panc-1, MIA PaCa-2, MCF7, and MDA-MB-231) and co-stained them with cytoskeletal α-Tubulin for confocal imaging (Figure 1H). In these cells, OpenEMMU enabled the evaluation of DNA replication and the simultaneous flow cytometric analysis of EdU with mitotic Histone H3 phosphorylated at serine 10 (p-HH3^S10^) and DNA damage (γH2A.X^S139^) markers (Figures S5B and S5D). Moreover, OpenEMMU effectively distinguished early, mid, and late S-phase cells based on the label intensity and characteristic genomic locations of DNA replication foci in cancer cell lines and hiPSCs (Figure 1H). We successfully co-stained 2-h EdU-labeled hiPSCs with the pluripotency transcription factor OCT3/4, reinforcing OpenEMMU’s versatility for co-immunolabeling (Figure S5E). To validate OpenEMMU’s accuracy in detecting DNA replication dynamically and measuring cell cycle progression, we stimulated DNA synthesis in C2C12 myoblasts and C3H10T1/2 mesenchymal stromal cells using FGF-2 and PDGF-BB recombinant ligands. Both growth factors induced DNA replication 3–4 times and promoted cell cycle progression in low-serum conditions, as measured by EdU incorporation and the horseshoe-shaped EdU/DNA bivariate fluorescence (Figure S5F). While these cell lines demonstrated increased cell cycle progression in response to both growth factors, C2C12 myoblasts exhibited more robust proliferation with FGF-2, whereas C3H10T1/2 cells responded more favorably to PDGF-BB (Figure S5F). This differential response is likely attributable to the distinct relative expression of PDGF receptor proteins (PDGFRα, PDGFRβ) in these cell types,^42^ highlighting the ability of OpenEMMU to provide nuanced data on cell signaling-driven cell cycle responses.

For our optimized OpenEMMU method, we calculated the cost per 0.5 mL of reaction for each reagent, including those required for fixation, permeabilization, and the click chemistry step with EdU-labeled cells. At the time of writing, the estimated cost for the OpenEMMU click chemistry reaction (approximately USD 0.09 per 0.5 mL), inclusive of wash buffer and associated steps, makes it approximately 143 and 169 times cheaper than the Invitrogen EdU Flow Cytometry kit and the VectorLabs kit (formerly Click Chemistry Tools), respectively (Figure 1I). Thus, our analysis demonstrates that OpenEMMU can be assembled in-house cost-effectively using common, off-the-shelf reactants and reagents.

### OpenEMMU demonstrates robust detection and cell cycle analysis compared to commercial 5-ethynyl-2′-deoxyuridine kits across diverse cell models

**Figure 2:**
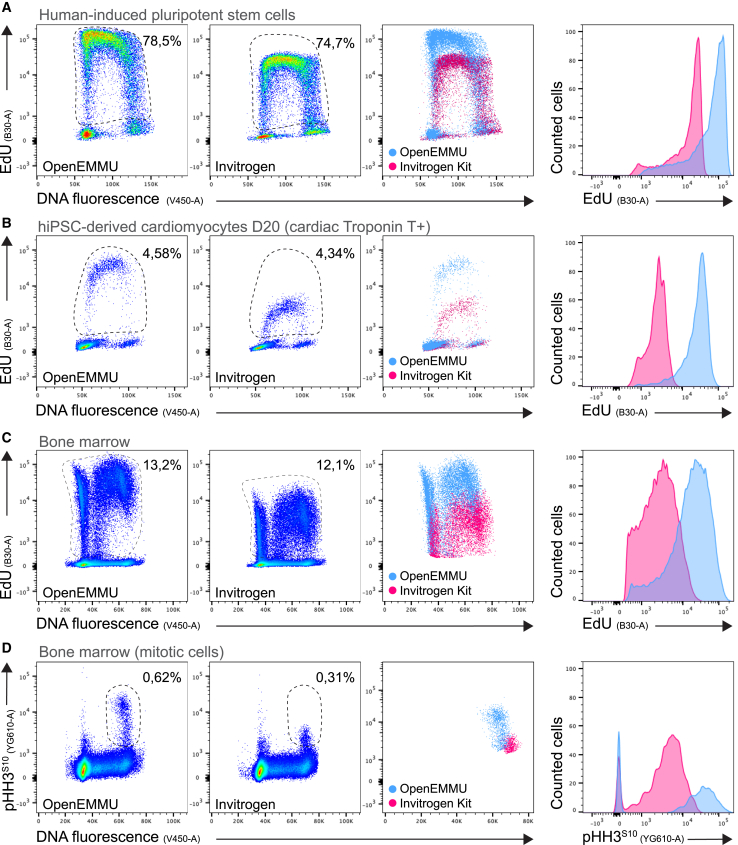
OpenEMMU outperforms commercial kits in evaluating DNA-replicating and mitotic cells (A) Flow cytometry results comparing OpenEMMU and the Invitrogen kit in terms of EdU fluorescence intensity and the proportion of EdU-labeled cells after a 2-h pulse in hiPSCs (*n* = 3). (B) Flow cytometry results comparing EdU fluorescence intensity and the proportion of EdU+ cells in hiPSC-derived cardiomyocytes (cTnT+) at day 20 of differentiation (*n* = 3). (C) Flow cytometry results comparing EdU fluorescence intensity and the proportion of EdU+ cells in total bone marrow cells of mice injected with EdU for 3 h (*n* = 3). (D) Flow cytometry analysis of phosphorylated histone H3 at serine 10 (p-HH3^S10+^) to detect mitotic cells after click chemistry using OpenEMMU versus the Invitrogen EdU kit, demonstrating a significant reduction in detected mitotic cells with the Invitrogen kit (*n* = 3).

We then compared directly the Invitrogen EdU kit for flow cytometry to OpenEMMU to quantitatively measure EdU fluorescence intensity and evaluate the accuracy of EdU fluorescence detection in S-phase cells. OpenEMMU consistently outperformed the Invitrogen Flow Cytometry kit in both aspects, accurately detecting a higher proportion of S-phase cells (by ∼5–10%, *n* = 3) and exhibiting ∼10-fold greater EdU fluorescence intensity (Figures 2A, 2B, and S6A). This superiority was evident across a wide range of cell types, including highly proliferative undifferentiated hiPSCs and slowly DNA-replicating cells such as hiPSC-derived cardiomyocytes at day 20 of differentiation and the C2C12 myoblast cell line (Figures 2A, 2B, and S6A).

Beyond cell lines, we compared the performance of OpenEMMU and Invitrogen EdU kits using tissue harvested after *in vivo* EdU uptake, including bone marrow cells and splenocytes from healthy wild-type mice. Adult WT male and female mice were intraperitoneally injected with EdU for 4 h (based on its bioavailability of approximately 1–2 h^43^), followed by OpenEMMU analysis of PFA-fixed cells. In bone marrow and spleen cells, OpenEMMU accurately stained DNA-replicating cells and produced significantly brighter EdU-labeled cells (i.e., fluorescence intensity) (Figures 2C and S6B). We then evaluated OpenEMMU’s compatibility with co-staining using a phycoerythrin (PE)-conjugated antibody to detect mitotic cells (p-HH3^S10+^). PE, a red protein pigment, is known to be adversely affected by CuAAC click chemistry in most commercial EdU kits,^44^ and as expected, detection of mitotic cells using a PE-conjugated antibody using the Invitrogen kit was suboptimal (Figure 2D). In contrast, OpenEMMU successfully labeled these cells, with a detection rate of 0.62% of mitotic cells compared to 0.31% using the Invitrogen EdU kit. Not only did the commercially available kit impact the proportion of p-HH3^S10+^ mitotic cells, but also the fluorescence intensity of the PE-conjugated antibody (Figure 2D). We further evaluated OpenEMMU against the Click-iT EdU Alexa Fluor 488 Imaging Kit and found that both methods yielded comparable performance levels in hiPSCs (Figure S6C). These results underscore OpenEMMU’s versatility across various cell models and staining procedures, making it a simple and adaptable EdU multiplexing methodology for studying DNA replication and the cell cycle.

### OpenEMMU enables multiplexing with conjugated antibodies for immune analysis

**Figure 3:**
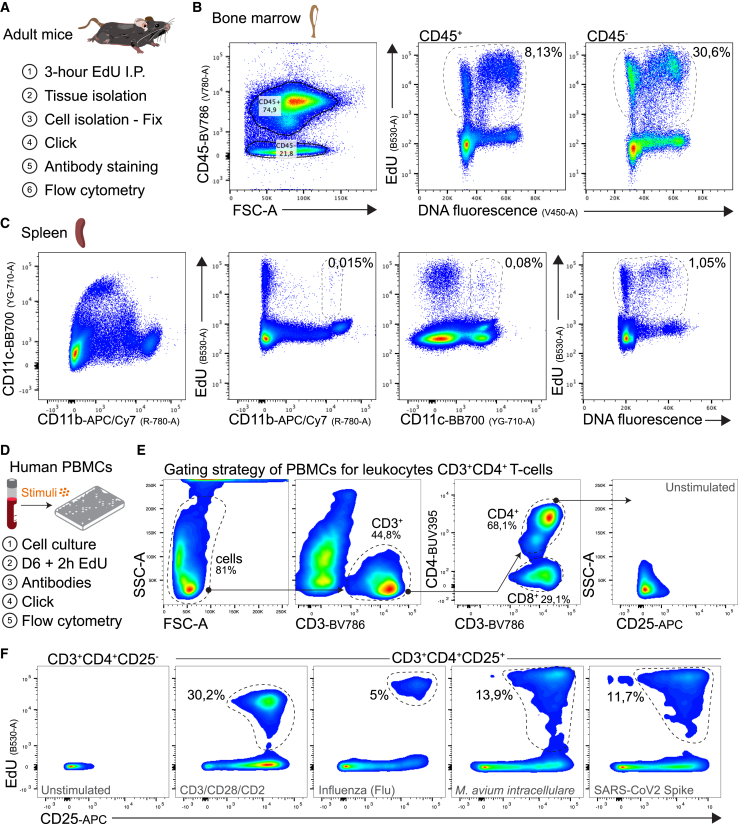
Compatibility of OpenEMMU for multi-parametric analysis of DNA replication in mouse and human immune cells (A) Outline of adult mice EdU injection and OpenEMMU protocol for studying DNA synthesis in various tissues and cells. (B) Flow cytometry of EdU signal using isolated bone marrow cells (*n* = 3). (C) Flow cytometry of EdU signal using isolated splenocytes, highlighting percentages of rare DNA replicating cells (CD11b+ and CD11c+) (*n* = 3). (D) Outline of freshly isolated human peripheral blood mononuclear cells (PBMCs) evaluation for EdU incorporation and responses to various immunological stimuli. (E) Gating strategy employed to detect DNA replication in activated human T-cells. The dashed lines indicate the selected gates. (F) Cell stimulation with activating antibodies or exposure to infectious disease-relevant immune stimulants showing the quantification of EdU+ DNA replicating CD3^+^CD4^+^CD25^+^ cells (dashed lines) (*n* = 2).

To challenge OpenEMMU’s multiplexing capabilities further, we first combined the detection of DNA-replicating bone marrow and spleen adult cells using EdU with immune markers such as CD45, CD11c, and CD11b, analyzed via flow cytometry (Figures 3A–3C and S7A). OpenEMMU effectively detected not only highly proliferative bone marrow cells but also splenocytes (CD11b/c^+^), known for their much lower proliferative rate–these were found to represent 1% of proliferative cells within the total splenocyte population (Figures 3B and 3C). Notably, OpenEMMU identified myeloid CD11b^+^ cells with an exceptionally low proliferation rate (0.015%) and CD11c^+^ classical DCs (0.08%) (Figure 3C).

To demonstrate OpenEMMU’s clinical relevance while extending its multiplexing scope, we next evaluated human T cell activation and CD3^+^CD4^+^ T cell proliferation using fresh peripheral blood mononuclear cells (PBMC) cultures. CellTrace Violet (CTV) labeled PBMC were cultured for 6 days using separate negative control cultures (medium only), positive control cultures with optimal T cell polyclonal antibody activation (CD3/CD28/CD2), as well as other cultures for proliferative responses to various recall antigens from infectious agents, including (i) influenza vaccine antigen; (ii) *M. avium intracellulare* lysate; and (iii) SARS-CoV-2 spike protein, respectively (Figures 3D–3F). In all tested conditions, proliferating EdU-labeled CD3^+^CD4^+^CD25^+^CTVdim T cells exhibited DNA replication in response to canonical CD3/CD28/CD2 stimulation and recall antigens, albeit at varying rates (Figure 3F). Our findings support earlier observations, confirming OpenEMMU’s multiplexing capabilities with various fluorochrome-conjugated antibodies. The simultaneous detection of EdU fluorescence alongside CD45, CD11b, CD11c, T cell lineage markers (CD3, CD4, CD8), T cell activation marker CD25, and cell division marker CTV, suggests that EdU-labeled, OpenEMMU-processed cells can be used to investigate additional cell markers across various cell types–a capability previously limited with commercial kits.

### 3D imaging of DNA replication and cycling cells in adult mice organs and iterative bleaching extends multiplexity imaging multiplexing

**Figure 4:**
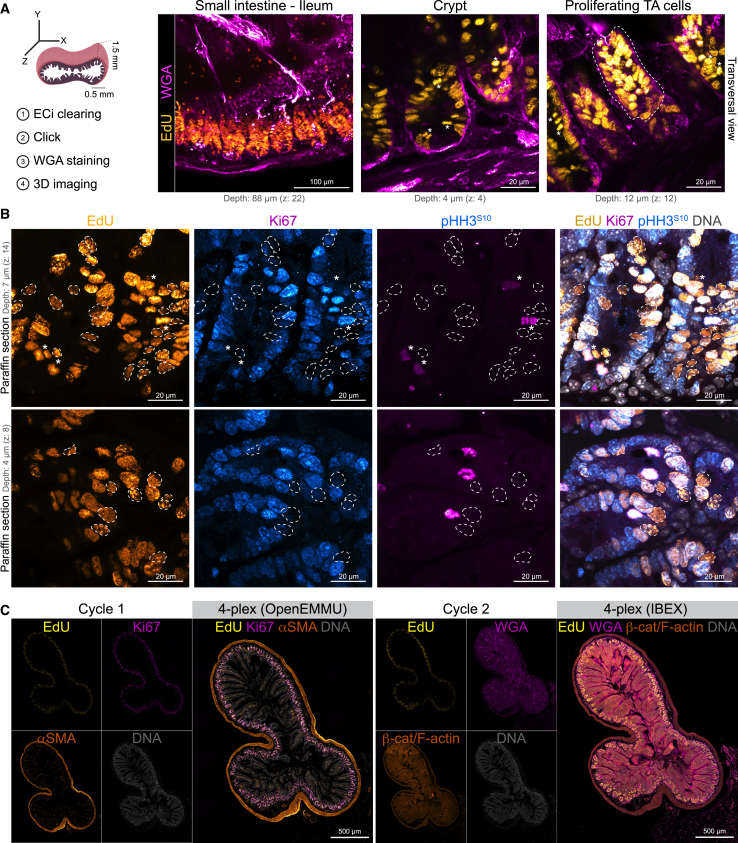
OpenEMMU for multiplexed imaging and DNA replication studies in the adult intestinal stem cell niche (A) OpenEMMU compatibility with ethyl cinnamate tissue clearing and 3D confocal laser imaging, during analysis of DNA synthesis in the small intestine (ileum) of adult mice injected with EdU for 4 h. Several proliferating cell regions are observed, including the adult stem cell crypt and the transient amplifying (TA) cells in the villi, highlighted by the dotted line. Asterisks indicate mitotic cells. (B) FFPE-processed ileum sections and confocal laser imaging of EdU in DNA-replicating cells, along with Ki67 and p-HH3^S10^ that are EdU positive, visualized by z stack confocal microscopy. Dotted strokes highlight EdU^+^ cells that are Ki67 negative. Asterisks indicate mitotic cells. (C) FFPE-processed ileum section, visualized using tiling confocal microscopy. The section underwent two cycles of 4-plex staining, with OpenEMMU used in the first cycle and IBEX applied in the second (*n* = 3).

Following the flow cytometric evaluation of OpenEMMU’s performance using dissociated single cells isolated from mice injected with EdU for 4 h, as described above, we applied the method to label cells in 3D mouse tissues, focusing first on the stem cell niche within the adult small intestine as a homeostatic proliferative and regenerative model. To achieve this, we combined OpenEMMU with ethyl cinnamate (ECi) tissue clearing. Laser confocal imaging of ECi-cleared ileum showed extensive EdU labeling of DNA-replicating cells across a region ∼100 μm in depth (Figures 4A, S7B, and S7C). This enabled targeted confocal microscopy at different focal planes to visualize specialized regions within the adult stem cell niche, such as the stem cell crypt, villus, WGA-enriched Paneth cells, and highly DNA replicating transient-amplifying cells (Figure 4A). We confirmed that either Saponin or Triton X-100-containing cell permeabilization/wash buffers could be used successfully in the OpenEMMU assay (Figure S7C). Lateral or top-view 3D imaging can be used for visualizing DNA replication and cycling cells (Figures 4A and S7B–S7D). Four hours of intraperitoneal EdU administration allowed for the successful identification of not only DNA-replicating cells but also cells that had progressed through the cell cycle (S and G2 phase) and reached mitosis, as judged by condensed chromosomes in prophase, metaphase, and anaphase (Figure 4A).

To further validate cell cycle progression assessment and immunolabelling compatibility of OpenEMMU, we stained formalin-fixed paraffin-embedded (FFPE) ileum sections using an optimized protocol for FFPE, deparaffinization, and antigen retrieval^45^ (Figures 4B and S7B). *In vivo* EdU labeling was extensively observed along the gut stem cell niche in highly DNA-replicating cells with the successful co-immunostaining of a proportion of cells with Ki67 (a nucleolar protein and proliferative capacity marker) and p-HH3^S10^ (a mitotic marker) (Figure 4B). DNA labeling was also achieved. We confirmed that only a few EdU-labeled cells had reached mitosis (p-HH3^S10+)^ during the labeling period. Furthermore, we observed instances where Ki67 staining appeared less intense than the EdU signal in actively cycling cells detected via OpenEMMU (dotted lines, Figure 4B). This suggested that a subset of EdU^+^ cells might lack strong Ki67 expression. To quantify this, we analyzed the co-expression and found that, on average, 31.7 ± 7.6% (mean ± SD, *n* = 7) of EdU-positive (EdU^+^) cells were indeed Ki67-negative (EdU^+^Ki67^Neg^) across the samples. This significant EdU^+^Ki67^Neg^ population may reflect both (i) the enhanced brightness and sensitivity of EdU detection using OpenEMMU, allowing the visualization of cells potentially missed by Ki67 staining, (ii) the highly heterogeneous dynamics of Ki67 protein abundance throughout the cell cycle, which can be low or absent during certain phases such as G0/G1 cells and S-phase entry/exit or transient cell cycle pauses, despite recent DNA synthesis.^46^^,^^47^ Consequently, OpenEMMU-EdU labeling is effective for capturing cells actively synthesizing DNA, including those with variable Ki67 levels.

To enable multiplexed imaging, we employed OpenEMMU, followed by iterative bleaching extends multiplexity (IBEX).^38^ IBEX is an open-source iterative immunolabelling and chemical bleaching technique that offers a low-cost and accessible approach using borohydride derivatives, requiring only basic laboratory skills.^48^ To assess the compatibility of OpenEMMU with this relatively novel multiplexing method, we first labeled FFPE ileum sections for Ki67 (R667), αSMA (Cy3), EdU (AZDye 488 Azide), and DNA (Vybrant Violet) during the first imaging cycle (Figure 4C). Following the completion of this round (cycle 1), we applied LiBH_4_ (1 mg/mL, 15 min incubation) to inactivate the fluorophores and proceeded with a second staining cycle (cycle 2), where we stained for WGA (CF640R) and β-catenin (PE) together with F-actin (Flash Phalloidin 594) (Figure 4C). Our observations revealed that EdU (AZDye 488 Azide) and Vybrant Violet (DNA staining) exhibited significant resistance to LiBH_4_ bleaching, as strong signals persisted even after two rounds of LiBH_4_ incubation. Consequently, re-labeling for EdU or DNA was unnecessary. A third immunolabelling cycle (cycle 3) was subsequently performed, staining for E-cadherin (A555) and COL1A1 (A555) simultaneously, and BODIPY 630/650 (Figure S7E). We noted that CF640R (WGA) also demonstrated partial resistance to LiBH_4_-mediated fluorophore inactivation, requiring longer bleaching (LiBH_4_, 2 × 15 min). Finally, we observed that the OpenEMMU-mediated click chemistry reaction is not affected by prior LiBH_4_ treatment (Figure S8). These findings show that OpenEMMU can be multiplexed to capture the *in situ* subcellular biology of complex tissues with precision and spatial resolution.

### Mapping DNA replication and cell proliferation in developing mouse embryos and organs

To further investigate the applicability of OpenEMMU to studying DNA replication and cell proliferation *in vivo*, we assessed (i) its ability to identify proliferative foci across other developing organs and tissues; (ii) the preservation of fine and fragile embryonic tissue structures in FFPE mouse embryos after deparaffinization, OpenEMMU click chemistry, and antibody staining; and (iii) its compatibility with complex 3D immunostaining using fluorochrome-conjugated and non-conjugated antibodies.

**Figure 5:**
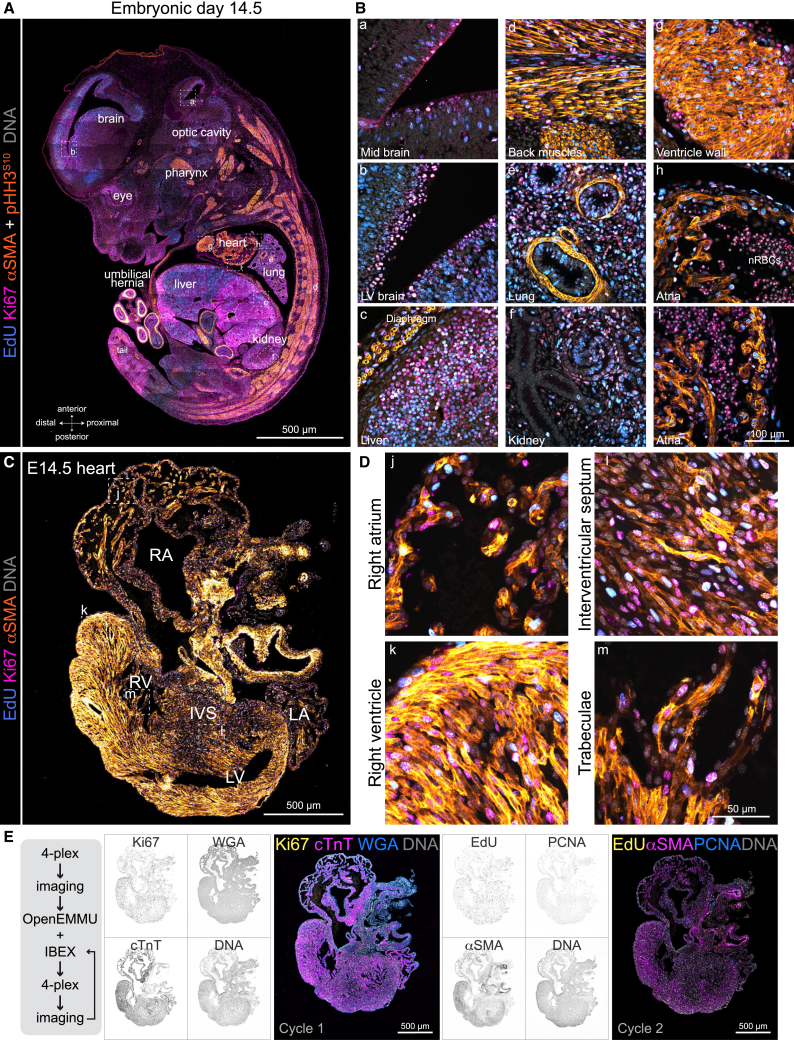
Embryo and organ-specific analysis of DNA replication and mitosis in developing mouse embryos (A) FFPE-processed embryonic day E14.5 mouse embryo and confocal tile imaging of EdU signal in DNA replicating cells multiplexed with Ki67, αSMA, phospho-histone H3^S10^, and DNA labeling. αSMA immunolabelling depicts the developing musculature, heart cardiomyocytes, as well as smooth muscle cells in multiple organs and tissues (*n* = 3). (B) Insets (B) show the magnification of the boxed areas using z stack confocal microscopy (a-i). (C) FFPE-processed E14.5 developing heart and confocal tile imaging of EdU signal in DNA replicating cells multiplexed with Ki67, αSMA, and DNA labeling (*n* = 2). αSMA immunolabelling depicts the developing musculature, heart cardiomyocytes, and smooth muscle cells in multiple cardiac regions. (D) Insets (D) show the magnification of the boxed areas using z stack confocal microscopy (j-m). (E) E14.5 heart section underwent two 4-plex staining rounds: first with conjugated antibodies and WGA, then with OpenEMMU and IBEX (*n* = 3).

To label developing embryos, pregnant females were administered a 3-h pulse of EdU. Using anti-Ki67, αSMA-Cy3, and p-HH3^S10^ immunohistochemistry, we imaged whole mouse embryo sections at E14.5 comprehensively, capturing the characteristic organ localization and organization using tile confocal microscopy and the Thunder Imager epifluorescence system for accelerated image acquisition (Figures 5 and S9). We observed EdU-positive cells with distinct nuclear labeling abundance, with different tissues and organs exhibiting varying degrees of DNA-replicating cells. Whereas some cells in specific organs and tissues such as the liver, lungs, and lateral ventricle (LV) of the brain were highly proliferative (higher EdU uptake), others, like those in the midbrain region, the developing ureteric bud region of the kidneys, and to a lesser extent developing skeletal muscles and heart, had a much-reduced proliferative rate (lower EdU uptake) (Figures 5A, 5B, and S9A). Next, we labeled earlier developing embryos at E11.5 with EdU for 3 h. OpenEMMU enabled the precise visualization of EdU^+^ cells alongside co-staining for Ki67, αSMA-Cy3, p-HH3^S10^, and DNA across various developing organs and tissues (Figures S9B and S9C). As evident in highly magnified regions, finer subcellular structures were well preserved throughout the deparaffinization and OpenEMMU procedures.

The adult four-chambered mammalian heart is the first organ to form and function in the embryo. To further elucidate DNA replication and cell proliferation in the developing mouse heart, we applied OpenEMMU. By E14.5, most cardiac structures have successfully developed, including the upper chambers (atria), distinct left and right ventricles, interventricular septum, transient outflow tract, and elements of the cardiac conduction system. Specific heart tissues and regions can be distinguished, such as the myocardium, epicardium, endocardium, coronary vasculature, trabeculae, and cushion tissues (Figures 5C and 5D). Using OpenEMMU in combination with immunohistochemistry against Ki67 and αSMA, we were able to focus and distinguish DNA-replicating and cycling cells in the developing heart, both αSMA^+^ and αSMA^−^ cells, enabling the faithful detection of DNA synthesis in cardiomyocytes and non-cardiomyocyte stromal cells (Figures 5C and 5D).

We then assessed whether OpenEMMU could be applied following an initial cycle of 3-plex immunostaining with DNA staining, followed by imaging, IBEX, OpenEMMU, and two additional IBEX cycles (Figure 5E). We successfully stained for Ki67-Vio-B515, WGA-CF555, cTnT-AF647, EdU-AZDye 488, PCNA-AF647, αSMA-Cy3, COL1A1-AF647, MLC2v-PE, MLC2a-APC, and p-HH3^S10^-PE, allowing the visualization of multiple spatial features within the developing heart (Figure 5E). Notably, Ki67 labeling was extensive, similar to PCNA, while EdU coverage was nuclear and more limited. Only a few rare mitotic cells (p-HH3^S10^) were observed, and these were primarily within the cardiac ventricles (MLC2v^+^) (Figures 5E and S9D). MLC2a specifically marked the developing left and right atria, whereas WGA staining was found throughout, strongest within the epicardium (Figures 5E and S9D). Thus, OpenEMMU can be effectively applied across ≥4 staining cycles, both before (Figures 4C and S9D) and after IBEX (Figures 4C and 5E), maintaining tissue and signal integrity.

**Figure 6:**
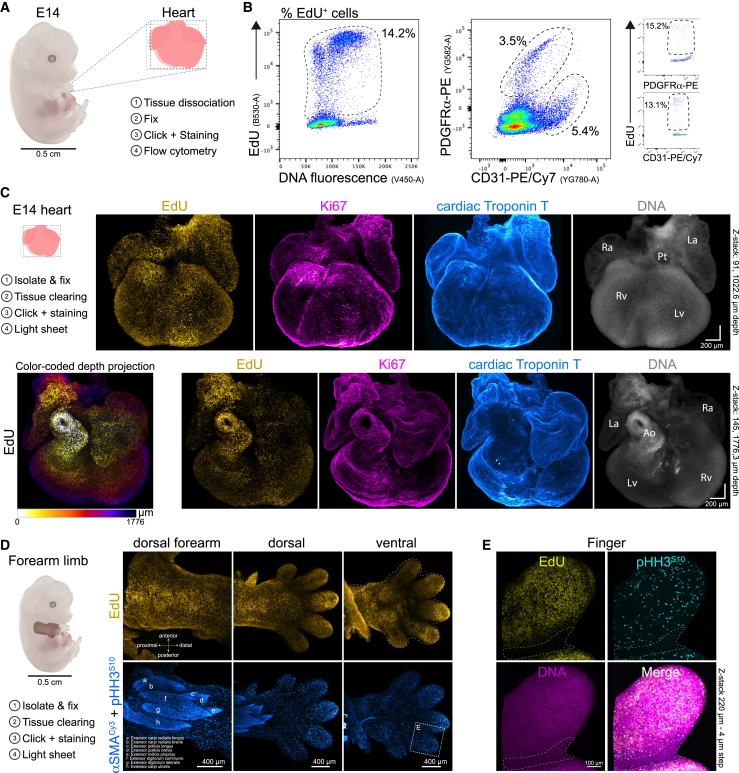
Application of OpenEMMU to complex developing organs and tissues (A) Diagram of an embryonic day 14 (E14) mouse embryo showing the steps involved in isolating the heart and the OpenEMMU protocol. (B) Flow cytometry results of isolated total heart cells with EdU staining along DNA content (left graph), demonstrating its multiplexing capability with CD31-PE/Cy7 and PDGFRα-PE. The relative proportion of EdU+ cells in both cell populations is depicted (*n* = 3). (C) Light-sheet fluorescence imaging of EdU multiplexed with Ki67 and cardiac Troponin T, along with DNA labeling in an E14 heart, shown from a front view (top panels) (*n* = 4). A top view of the same heart is provided, with EdU labeling color-coded by depth projection. Ra, right atrium; La, left atrium; Pt, pulmonary track; Rv, right ventricle; Lv, left ventricle; Ao, aorta. (D) Light-sheet imaging of EdU multiplexed with αSMACy3 and p-HH3^S10^-A594 immunolabeling, along with DNA staining of an E14 forelimb, shown from dorsal and ventral views (*n* = 3). (E) Inset of (D, ventral side of the digit) showing a magnified view of the boxed area using z stack confocal microscopy. The dotted lines indicate the interdigital necrotic zone, where cellular DNA synthesis and mitosis decrease dramatically.

To investigate whether OpenEMMU could be used to profile individual cells and their cell cycle accurately and quantitatively during heart development, we first isolated E14-developing hearts and performed immunolabeling against PDGFRα (fibroblasts) and CD31 (endothelial cells), along with detecting EdU^+^ cells by flow cytometry (Figure 6A). The visualization of these distinct populations of cells showed that approximately 13% of fibroblasts and 15% of endothelial cells were stained with a 3-h EdU pulse (Figure 6B). Confocal imaging-based assessment of total dissociated WGA-stained cells demonstrated that approximately 12% were EdU^+^ (Figures S10A–S10C). We were able to profile both stromal cells and cardiomyocytes expressing cardiac muscle Troponin T (TNNT2, also known as cTnT), which exhibited strong EdU labeling at this developmental stage (Figure S10D). Together, these results establish that OpenEMMU is compatible with a spectrum of staining procedures for which existing methods are not fully optimized.

### Mapping DNA replication and cell proliferation in whole-organ 3D fluorescence imaging

Due to the limitations of commercial EdU kits in permeabilizing and penetrating complex 3D organs and structures, sensitive 3D imaging is often limited. We sought to investigate the applicability of OpenEMMU for detecting DNA-replicating cells in whole developing organs, including the heart and limbs. Using DEEP-Clear tissue clearing^49^ and light-sheet fluorescence microscopy (LSFM) on a developing mouse heart at E14, we achieved the 3D visualization of major mammalian cardiac structures through immunohistochemistry against cardiac Troponin T, highlighting extensive EdU labeling at this developmental stage (Figure 6C). Notably, EdU labeling outperformed Ki67 in detecting cells in the S-phase of the cell cycle, as areas containing EdU-labeled nuclei were often only partially positive for Ki67 (Figure 6C). This observation is consistent with our previous results using tissue sections (Figure 4B).

To further explore OpenEMMU 3D imaging capabilities, we evaluated EdU labeling in an E14 (TS21 to TS22+) forearm limb using LSFM (Figure 6D). We observed extensive DNA synthesis, as indicated by EdU staining, and co-detected rounded, chromatin-dense mitotic cells using a PE-conjugated antibody against p-HH3^S10^ (Figures 6D and S10E). OpenEMMU, in combination with αSMA-Cy3 immunostaining, enabled the visualization of distinct developing extensor muscle groups of the forearm with myofiber resolution (Figures 6D and S10E).^50^ EdU-labeled cells and mitotic cells could also be distinguished with single-cell resolution using not only LSFM but also confocal microscopy (Figures 6D and 6E). Notably, a detailed YZ-axis view of light-sheet images of the developing forearm extensor muscles showed labeling at imaging depths of over 150 μm (Figure S10F). These results demonstrate the extended applicability and scalability of OpenEMMU to more complex organs and tissues, preserving morphological features and staining quality.

### Profiling DNA replication and cell proliferation in 3D self-organizing cardiac organoids

iPSC and embryonic stem cell-derived organoids have revolutionized our understanding of tissue and organ morphogenesis, as well as cell fate and behavior.^51^ Organoids serve as invaluable research platforms for investigating cell specification, organ growth, and maturation within complex and physiologically relevant 3D environments, heralding significant advancements in biomedical research. Given the current challenges in accurately modeling early human heart development, 3D cardiac organoids derived from human iPSCs present a promising platform for studying cardiac development and congenital heart disease,^52^^,^^53^ which affects approximately 1% of newborns worldwide.^54^

**Figure 7:**
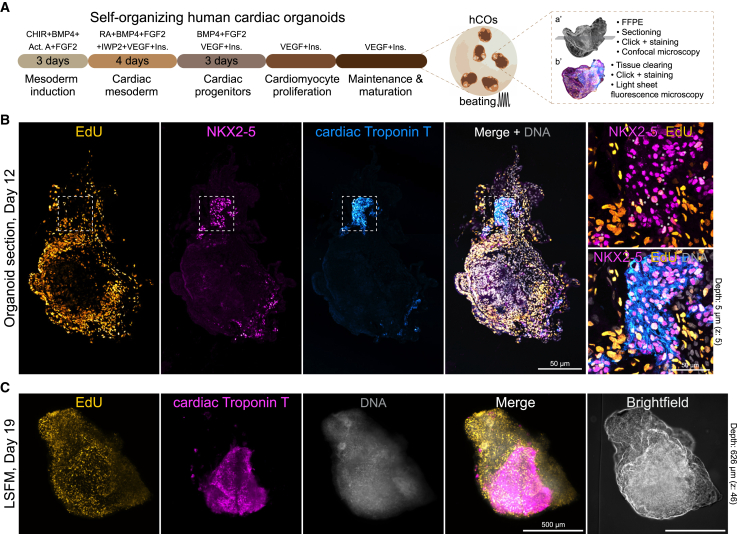
DNA replication in self-organizing 3D human cardiac organoids (A) Protocol outline to generate self-organizing and beating hiPSC-derived human cardiac organoids (hCO), showing two distinct imaging strategies: FFPE organoid confocal imaging (a’) and 3D light sheet microscopy (b’). (B) FFPE-processed day 12 organoid and confocal tile imaging of EdU fluorescence in DNA replicating cells (*n* = 6). NKX2-5 and cTnT expressing cardiac progenitors are highlighted in the boxed area. NKX2-5 and EdU double-positive cells are also shown (∗, magnified box). (C) Whole-mount light sheet microscopy (LSFM) 3D visualization of a hCO at day 19 showing EdU positive cells and cTnT expressing cells. DNA-labelled nuclei are also shown (*n* = 6).

We evaluated OpenEMMU’s performance in labeling and visualizing self-organized hiPSC-derived cardiac organoids (hCOs) at days 12 and 19 of differentiation and early maturation (Figure 7), observing effective staining of proliferative cells within beating organoids, albeit at varying intensities across distinct regions (Figures 7B and S11). Overall, we observed that the majority of internal regions exhibited a weak EdU label. This may be attributed to slower cell cycle progression in comparison to cells nearer the hCO’s periphery (media boundary), likely due to cell differentiation, tissue maturation, or diminished tissue perfusion (Figures 7B and S11). When co-staining for the NKX2-5 cardiac transcription factor, we noted that several NKX2-5-expressing cardiomyocytes were also positive for EdU, while others were not (Figure 7B). These cells were also embedded within cardiac Troponin T-expressing regions, confirming their cardiac and sarcomeric contractile identity (Figures 7B and S11).

Because of the challenges associated with using FFPE for relatively miniature and fragile organoids, we optimized an OpenEMMU labeling and organoid-clearing strategy to visualize complex 3D structures within hCOs better using LSFM. As previously observed, OpenEMMU was compatible with the staining of 3D structures. Resulting images helped identify cardiac Troponin T^+^ regions with depths exceeding 500 μm (Figure 7C). Additionally, strong EdU staining was also observed in the non-cardiac areas (Figure 7C). Together, our results demonstrate that OpenEMMU, in combination with immunofluorescence, can be used to gain a deeper understanding of DNA replication and cell proliferation in human developing organs and tissues. Moreover, it is effective in organoid-derived platforms, with the potential to offer insights into cell proliferation in both health and disease.

### Analysis of DNA replication in zebrafish larvae

Zebrafish are known for their small size, rapid external development, imaging clarity, and low-cost husbandry, making them a valuable model for vertebrate development and human research.^55^^,^^56^ Zebrafish embryos and larvae have been effectively utilized to investigate the biological impact of a variety of compounds, drugs, and chemicals on different organs and tissues.^57^^,^^58^ They complement mammalian models, offering advantages for large-scale genetic studies and rapid gene function analyses.

**Figure 8:**
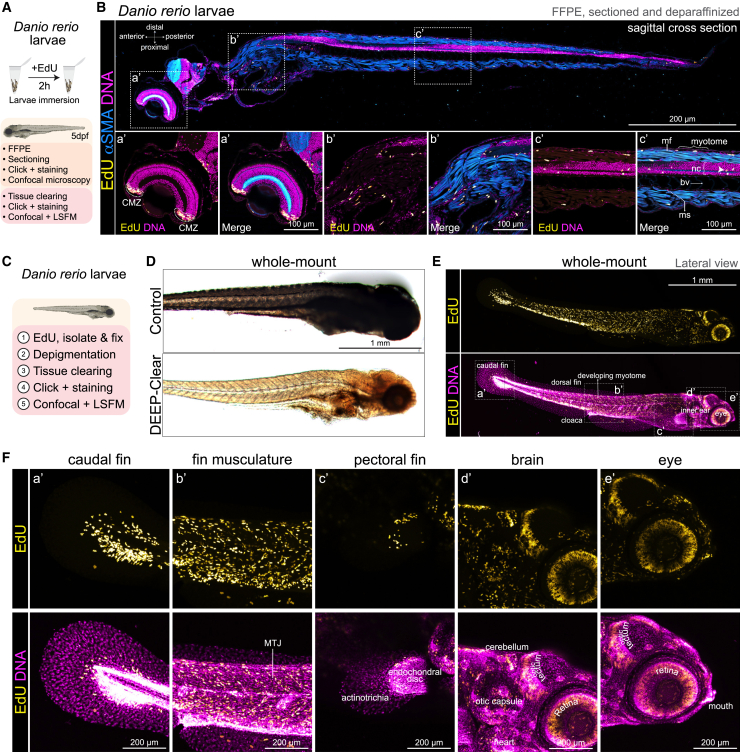
Analysis of DNA replication in whole zebrafish larvae (A) *Danio rerio* 5dpf larvae immersion protocol aimed at labeling DNA synthesis with EdU for 2h using FFPE (B) or whole-mount staining (D–F). (B) FFPE-processed larva sagittal cross section and confocal tile imaging of EdU signal in DNA replicating cells (*n* = 3). αSMA-Cy3 immunolabeling depicts the developing musculature and growing myotomes. Insets (below) show the magnification of the boxed areas (a’-c’). Arrowhead shows a mitotic event. (C and D) Depigmentation and DEEP-Clear protocol allow the whole-mount visualization of growing larvae (*n* = 6). (E and F) EdU and DNA labeling revealing highly proliferative cells, visualized by tiling (E) and z stack (F) confocal microscopy. (F) Insets (F) show the magnification of the boxed areas (a’-e’). CMZ, ciliary marginal zone; MTJ, myotendinous junction; nc, notochord; ms, myoseptum; mf, myofiber; bv, blood vessels.

Following a 2-h EdU immersion protocol of *Danio rerio* larvae 5 days post-fertilization (dpf), OpenEMMU facilitated a detailed anteroposterior overview of DNA synthesis using FFPE samples, in combination with αSMA immunostaining and DNA staining (Figures 8A, 8B, and S12A–S12C). By 5 dpf, zebrafish larvae are free-swimming and independent feeders, although many of their organs and structures, such as the caudal and pectoral fins, developing musculature, brain, eyes, and jaws, continue to develop.^59^ EdU-labeled cells were detectable in highly proliferative regions across the developing larvae, including the ciliary marginal zone (CMZ) in the developing teleost retina,^60^ the developing myotome, and notochord (Figure 8B). Sporadic mitotic chromosomes were identified using OpenEMMU (Figure 8B, magnified regions), demonstrating that EdU-labelled cells can progress from the S phase to the G2 and M phases of the cell cycle in 5dfp developing larvae. These results were obtained using either confocal laser microscopy or the Thunder Imager epifluorescence system for faster data acquisition (Figures 8B and S12C).

Having confirmed OpenEMMU’s efficacy in the 3D staining of whole organs in mice and iPSC-derived organoids, we combined *in vivo* labeling of nascent DNA using whole-organism tissue clearing (i.e., DEEP-Clear^49^) to comprehensively visualize DNA replication activity in 5dpf zebrafish larvae (Figure 8C). Depigmentation and 3D tissue clearing enabled the whole-mount visualization of the 5dpf zebrafish larvae and facilitated OpenEMMU for effective 3D nascent DNA visualization across the entire organism (Figures 8D, 8E, and S12D) and at different focal planes, as observed with either confocal laser microscopy or LSFM (Figures 8E, S12E, and S12F). EdU-labeled cells were extensively observed in the growing caudal fin and the distal portion of the endochondral disc of the pectoral fin (Figure 8F). They were also present across the developing myotome, including the myotendinous junctions (Figure 8F). Additionally, these cells were found in a cellular structure on the lateral surface of the myotome known as the external cell layer (ECL) (Figure 8F), which is responsible for generating stem and progenitor cells for larval muscle growth.^61^ Distinct regions of the larvae’s head, such as the cerebellum, the optic tectum, and the otic capsule, also showed substantial EdU labeling (Figure 8F). Additionally, extensive DNA replication was observed in the developing retina and mouth (Figures 8F and S12F). Finally, we visualized regenerative adult zebrafish ventricular resections using OpenEMMU. EdU-labeled cells were observed throughout the trabeculated myocardium, with enrichment in the regenerating ventricular apex, as anticipated (Figure S12G).

### DNA replication stress during gastrulation impairs zebrafish embryogenesis

**Figure 9:**
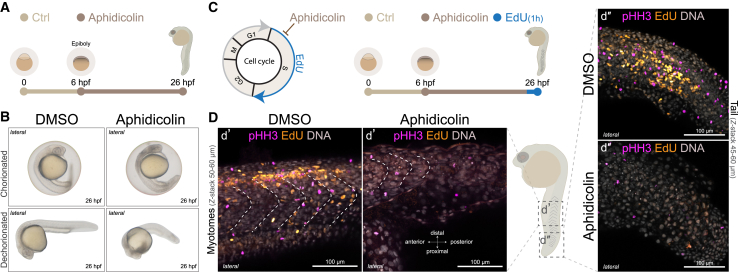
Global DNA replication stress impairs zebrafish embryogenesis (A) Schematic representation of the experimental workflow. *Danio rerio* embryos were treated with aphidicolin, a DNA polymerase inhibitor, starting at 6 h postfertilization (hpf) to inhibit embryo-wide DNA replication during S-phase. (B) Bright-field images of representative embryos collected at 26 hpf, shown with the chorion intact (top) and removed (bottom) (*n* = 10). (C) To assess DNA synthesis and cell proliferation under replication stress, dechorionated embryos were immersed in EdU for 1 h during aphidicolin treatment, followed by fixation and OpenEMMU. (D) Whole-mount imaging of depigmented and optically cleared embryos enabled the high-resolution visualization of proliferative activity (EdU incorporation) across embryonic tissues (*n* = 10). Confocal z stack images show EdU incorporation, phospho-Histone H3 (Ser10) staining (pHH3) for mitotic cells, and nuclear DNA labeling. Insets (d′–d″) highlight magnified views of the boxed regions. The dotted lines indicate the developing myotomes, which are forming into segmented blocks of muscle.

The proper coordination of DNA replication and cell division is essential for cell differentiation and embryonic development. However, the impact of global replication stress on early vertebrate embryogenesis remains poorly understood. To address this question, we investigated how the pharmacological inhibition of DNA replication affects cell proliferation and morphogenesis in developing zebrafish embryos using OpenEMMU. We treated zebrafish embryos with aphidicolin, a potent B-class DNA polymerase inhibitor,^62^ beginning at 6 h postfertilization (hpf) to induce global replication stress from early gastrulation and evaluate its impact in vertebrate development (Figure 9A). Bright-field imaging at 26 hpf revealed that aphidicolin-treated embryos exhibited developmental abnormalities compared to untreated controls, including delayed eye and head morphogenesis, fragility, and altered body axis formation and elongation (Figure 9B). These underdeveloped phenotypes were evident both with the chorion intact and after manual dechorionation (*n* = 10 embryos per group), aligning with recent findings using a combination of two major DNA replication stress inducers, aphidicolin and hydroxyurea.^63^

To assess the effects of replication stress on DNA synthesis and mitotic progression, we performed EdU uptake and phospho-Histone H3 (pHH3) immunostaining assays using OpenEMMU of depigmented and optically cleared embryos, enabling the whole-mount visualization of proliferative activity across embryonic tissues. Embryos were exposed to EdU for 1 h during the final phase of aphidicolin treatment (Figure 9C). The administration of aphidicolin led to a pronounced reduction in DNA replication and mitotic activity, as evidenced by the near absence of EdU-positive cells and a substantial decrease in pHH3-positive mitotic cells in treated embryos (Figure 9D). Reduced trunk and tail tissue organization was concomitant with relatively abundant small and condensed DNA-rich structures, likely corresponding to apoptotic bodies (Figure 9D). These findings demonstrate that global replication stress severely impairs cell proliferation and organogenesis and highlight the utility of the EdU labeling of whole-mount cleared specimens for assessing DNA replication dynamics *in vivo* using OpenEMMU.

## Discussion

### Development and optimization of clickable OpenEMMU to measure DNA replication and cell-cycle progression

We report the development and optimization of a robust and open-source toolkit for DNA replication profiling and cell cycle measurement using CuAAC click chemistry. OpenEMMU facilitates the efficient detection of EdU incorporation in cultured cells, tissues, organs, and entire organisms using defined and cost-effective click chemistry reagents, significantly advancing the detection and analysis of DNA synthesis across various cell types and models, including human cells. We sequentially analyzed the influence of each chemical reagent to determine the conditions required for optimal labeling efficiency, as well as the quality and intensity of EdU fluorescence. Our systematic approach in refining the EdU click reaction demonstrates that OpenEMMU surpasses the performance of commercially available kits, particularly in terms of cost-effectiveness, versatility, and signal detection.

The key optimizations, including the determination of 800 μM as the crucial reagent concentration for CuSO_4_ in the OpenEMMU assay, underscore the importance of fine-tuning reaction components to achieve high-efficiency labeling and a superior signal-to-noise ratio. This adjustment, along with the in-house optimization of the permeabilization/wash buffer, serum, L-ascorbic acid, and picolyl azide concentrations, has resulted in a highly sensitive and specific custom assay capable of accurately distinguishing between various phases of the cell cycle and measuring DNA replication in different cellular contexts. The substantial improvement in EdU signal intensity (∼10-fold) compared to the Invitrogen commercial kit further validates the efficacy of OpenEMMU, particularly in scenarios requiring the co-detection of DNA synthesis and other cellular markers, PE-conjugated antibodies, IBEX-mediated imaging multiplexing, and complex 3D organs and organisms.

### The broad applicability of OpenEMMU across different cell types, organs, and tissues

OpenEMMU’s performance and its ability to sensitively detect DNA replication across a spectrum of cellular and molecular contexts, from proliferative hiPSCs, C2C12, C3H10T1/2, pancreatic and breast cancer cell lines, and growth factor-stimulated cell proliferation to low-replicating hiPSC-derived cardiomyocytes, activated human T cells, and *in vivo* murine cells (e.g., small intestine, bone marrow, and spleen), showcases its broad applicability, robustness, and adaptability. The method’s compatibility with fluorescence imaging, flow cytometry, and FACS should enhance researchers’ ability to dissect cell cycle dynamics in complex biological problems with high precision and combine OpenEMMU with genomics and spatial-’omics approaches. Moreover, the ability to multiplex EdU detection with various immunolabeling techniques, including the detection of cell surface markers and intracellular and nuclear proteins, enhances its utility in studies of cell identity, cell differentiation, organismal development, and disease.

One of the OpenEMMU’s most compelling advantages is its resource efficiency. By utilizing commonly available laboratory reagents, OpenEMMU offers a highly economical and robust alternative to commercial kits, costing approximately USD 0.05 per 1 mL of clickable solution–170 times cheaper than Invitrogen and 140 times cheaper than VectorLabs kits. This makes sophisticated DNA replication studies more accessible to diverse scientific communities. We have provided step-by-step imaging and flow cytometry protocols to guide the OpenEMMU user through the various protocols, making it labor-efficient. Its affordability and user-friendliness do not come at the expense of performance; rather, OpenEMMU outperforms commercial kits in several key aspects, including signal intensity, compatibility with PE-conjugated and non-conjugated antibodies, and the ability to label a wide range of cell types and complex 3D tissues and structures. Our open-source philosophy should allow for further advancements. Its compatibility with iterative labeling methods, such as IBEX, further enhances its utility for comprehensive biological studies, positioning it to revolutionize multi-target fluorescence research.

### OpenEMMU in multiplexed immunological and human T cell activation and proliferation

We demonstrate OpenEMMU’s utility as an adaptable platform for multiplexed analysis in immunological studies, extending its application to both homeostatic immune cell DNA replication and antigen-driven T cell activation and proliferation (Figure 3). PBMC-based assays offer a practical *ex vivo* approach to model the complex interplay of antigen presentation and T cell activation, and are increasingly leveraged for assessing the immunogenicity of therapeutic antibody candidates.^64^ This mechanism involves stimulating PBMCs, enabling antigen-presenting cells to process and present antigenic peptides, thereby initiating naive T cell activation, proliferation, and cytokine production. While flow cytometry with cell tracer dyes (e.g., CFSE or CellTrace Violet dilution) is a common method for evaluating antigen-specific T cell proliferation, the potential of EdU assays for directly detecting DNA replication has been less fully exploited. Furthermore, combining proliferation assessment with the analysis of T cell activation markers such as CD25 is crucial for gaining deeper insights into the activation state and the overall strength of the T cell response.^65^ Leveraging these capabilities, OpenEMMU offers a robust platform for exploring therapeutic-specific antibody responses and evaluating the immunogenicity of therapeutic antibody candidates.

Notably, this is the first known application of the EdU thymidine analog to assess human T cell activation and DNA replication in response to several physiologically relevant stimuli,^66^^,^^67^ including common anti-CD3/anti-CD28/anti-CD2 polyclonal activators, influenza vaccine, *M. avium intracellulare*, and recombinant trimeric SARS-CoV-2 spike protein. OpenEMMU’s ability to simultaneously detect DNA replication and a variety of surface markers in human T cells creates new opportunities for in-depth investigations into lymphocyte immune responses under typical and pathogenic challenges. Looking forward, OpenEMMU should help unveil new perspectives on immune cell populations and functional states in both health and disease, such as within tumor microenvironments or autoimmune conditions.

### OpenEMMU for whole-organ, organoid, and whole-organism 3D fluorescence imaging of nascent DNA

The application of OpenEMMU in 3D tissue imaging and whole-organ studies–such as mouse embryonic development, heart and forelimb development, and zebrafish–opens new avenues for exploring DNA replication in complex biological systems. This is achieved using cutting-edge technologies, including tissue clearing (e.g.,^68^^,^^69^ STAR Methods) and advanced imaging techniques such as light-sheet fluorescence microscopy.^70^ The ability to map DNA replication in 3D in developing organs, particularly in spatially challenging contexts such as heart development, whole-mount zebrafish larvae, and self-organizing cardiac organoids, is a significant advancement, and this capability will facilitate the understanding of organ and tissue regeneration. For instance, in cardiac organoids, we observe varied co-staining patterns of EdU with cardiac markers NKX2-5 (cardiac progenitors/early differentiating cardiomyocytes) and Troponin T (maturing cardiomyocytes). These patterns are consistent with the dynamic processes of organoid development, where proliferation primarily occurs in progenitor populations, with more limited division in differentiated cell types and non-myocyte populations. This qualitative observation validates OpenEMMU’s ability to identify proliferative cells within phenotypically defined populations, aligning with the established understanding of human cardiac organoid cellular heterogeneity and differentiation.

OpenEMMU allowed us to distinguish varying degrees of DNA replication, revealing significant differences and proliferative foci across multiple organs and cell types. Our results demonstrate that OpenEMMU addresses critical methodological limitations in studying DNA replication dynamics during vertebrate development. The ability to visualize EdU incorporation and mitotic activity across entire cleared zebrafish embryos provides unprecedented spatial resolution of replication stress effects that conventional sectioning approaches cannot achieve (Figure 9). This comprehensive visualization capability is particularly valuable for understanding how cellular processes such as DNA replication coordinate with morphogenetic movements during organogenesis. The approach established here opens new avenues for investigating replication stress in disease models and regenerative processes, where understanding tissue-wide proliferative dynamics is essential for mechanistic insights. The 3D mapping of DNA replication activity as it correlates with cell division rates, transcriptional activity, and metabolic processes, will deepen our understanding of the complexities of growth, function, and morphology in developing organisms.

OpenEMMU has proven to be fast, efficient, affordable, and scalable. These advances are expected to significantly influence future research on cell-cycle progression in various cell types through cost-effective, open-source assays, including the validation and extension of previous findings using thymidine analog uptake (e.g., BrdU, EdU). CuAAC click chemistry also has multiple biomedical and non-biomedical applications, including radiochemistry,^71^ nucleic acid,^13^ RNA,^72^ glycan,^73^ lipid,^74^ *in vivo* drug binding,^20^ and protein biology.^75^ Thus, open-source and reliable click chemistry methods are benefiting the development of custom assays and novel techniques in biotechnology, biomedicine, biochemistry, nanotechnology, and materials science.

In summary, our study introduces OpenEMMU, an open-source toolkit for DNA replication profiling and cell cycle analysis using CuAAC click chemistry. This dual modality enhances the sensitivity and resolution of DNA synthesis assessments and simplifies experimental workflows, representing a substantial advancement over existing protocols. By optimizing key reagents such as CuSO_4_, ascorbic acid, and picolyl azide, we achieved improved, robustness, and more reliable CuAAC click chemistry compared to standard commercial kits. We successfully integrated OpenEMMU with tissue depigmentation, custom cell permeabilization, blocking protocols, multiplex imaging using IBEX, and advanced tissue-clearing techniques such as DEEP-Clear. Validated in various 2D cell cultures and complex 3D structures, OpenEMMU offers reproducible workflows for efficient DNA replication characterization without the need for commercial kits. This platform enhances scalability, significantly reduces costs, and supports researchers across diverse models and systems. OpenEMMU has broad applications in cancer biology, immunology, stem cell and developmental biology, pathophysiology, and disease modeling, with the potential to become a standard technique for DNA replication and cell cycle research.

### Limitations of the study

Our research concentrated on utilizing Cu(I)-Catalyzed Azide−Alkyne Cycloaddition (CuAAC) click chemistry to monitor and assess DNA replication using flow cytometry and microscopic analyses. It is crucial to acknowledge that other click chemistry reactions or azide combinations (e.g., biotin azide) may demonstrate different efficiencies and labeling characteristics. Additionally, biases or technical limitations could arise from variations in experimental design, such as pulse-chase experiments, labeling duration, and the reagents employed. Therefore, significant new insights into cell cycle dynamics will necessitate validation across multiple laboratories and through diverse methodologies.

## Resource availability

### Lead contact

Requests for further information and resources should be directed to and will be fulfilled by the lead contact, Osvaldo Contreras (o.contreras@victorchang.edu.au).

### Materials availability

All materials used in this study are publicly available. This study did not generate new materials.

### Data and code availability

This article does not report original code. All data needed to evaluate the conclusions in the article are present in the article and/or the supplemental information. Any additional information required to reanalyze the data reported in this article is available from the lead contact upon request.

## Acknowledgments

We are grateful to Sarah Hancock and Nigel Turner for providing the Panc-1, MIA PaCa-2, MCF7, and MDA-MB-231 cancer cell lines, and to Dawei Zheng and Emily Wong for supplying wild-type 5dpf zebrafish larvae, as well as Alvaro Rajal for supplying zebrafish embryos. We also thank Kazu Kikuchi for the slides of injured adult zebrafish hearts and Bernice Stewart for administrative support. Special thanks to Michael Lovelace, manager of the Live Imaging Facility (LIF) at St. Vincent’s Center for Applied Medical Research (AMR), for his invaluable advice and assistance with the Leica Thunder microscope, and to the members of the Harvey Lab for their feedback. We appreciate Cecelia Jenkin and Aaron Hay for their zebrafish husbandry, the VCCRI BioCORE facility members, Scott Page for his guidance on the Lightsheet Z.1, and the 10.13039/100007221Victor Chang Cardiac Research Institute Innovation Center (funded by the 10.13039/501100021708New South Wales Government 10.13039/100009647Ministry of Health). We also thank the Garvan Weizmann Center for Cellular Genomics (GWCCG), the Core Facility Flow and Histopathology Facilities at the 10.13039/501100003355Garvan Institute of Medical Research (GIMR) for their infrastructure support, particularly Eric Lam, Anaiis Zaratzian, and Andrew Da Silva. Most figures were created using Adobe Illustrator and Adobe Photoshop 2024, Adobe. Figures S2 and S3 were created with BioRender.com. The graphical abstract was developed in collaboration with Illustrascilab (www.illustrascilab.com). Artwork was created by Felipe G. Serrano. This work was supported by grants from the 10.13039/501100000925National Health and Medical Research Council (NHMRC) of Australia (Investigator Grant (L3) GNT2008743; 10.13039/100019768Ideas Grant GNT2000615 to R.P.H.), the Medical Research Future Fund (MRFF) Genomics Health Futures Mission (2016033), 10.13039/501100021708NSW Government 10.13039/100009647Ministry of Health (Cardiovascular Disease Senior Scientist Grant to R.P.H.), 10.13039/501100000923Australian Research Council (ARC) (DP210102134), 10.13039/100007221Victor Chang Cardiac Research Institute Innovation Center (funded by the 10.13039/501100021708New South Wales Government 10.13039/100009647Ministry of Health), Miltenyi Research Award 2022 (to O.C.), the Medical Research Future Fund - MRFF Stem Cell Therapies Mission (2024/MRF2032746 to O.C.) and 10.13039/100007221Victor Chang Cardiac Research Institute (Outstanding Early and Mid-Career Researcher Grant to O.C.). R.P.H. held an 10.13039/501100000925NHMRC Senior Principal Research Fellowship (GNT1118576), and N.J.M. held an 10.13039/100015539Australian Government Training Program Scholarship.

## Author contributions

Conceptualization: O.C. Methodology: O.C., C.T., J.Z., N.J.M., I.A.M., A.G.C. Validation: O.C., C.T., J.Z., N.J.M., I.A.M., A.G.C., R.P.H. Formal analysis: O.C, C.T. Investigation: O.C., C.T., J.Z., N.J.M., I.A.M. Resources: O.C., J.Z., A.G.C., R.P.H. Supervision: O.C., R.P.H. Data curation: O.C., C.T., J.Z. Project administration: O.C. Funding Acquisition: O.C., R.P.H. Writing–original draft: O.C. Writing–review and editing: All authors. All authors discussed the results and revised the article.

## Declaration of interests

The authors declare that they have no competing interests.

## STAR★Methods

### Key resources table

**Table:** 

REAGENT or RESOURCE	SOURCE	IDENTIFIER
**Antibodies**		

Alexa Fluor® 647 anti-Tubulin-α, Immunofluorescence (1:250)	BioLegend	Cat. No. 627908; RRID:AB_2563178
PE anti-Histone H3 Phospho (Ser10), Immunofluorescence (1:250)	BioLegend	Cat. No. 650807; RRID:AB_2564562
Alexa Fluor® 594 anti-Histone H3 Phospho (Ser10), Immunofluorescence (1:200)	BioLegend	Cat. No. 650809; RRID: AB_2801122
Nucleolin (D4C7O) Rabbit mAb, Immunofluorescence (1:500)	Cell Signaling Technology	Cat. No. 14574; RRID:AB_2798519
Oct-3/4 (H-134), Immunofluorescence (1:250)	Santa Cruz Biotechnology	Cat. No. sc-9081; RRID:AB_2167703
Purified Rat Anti-Mouse CD16/CD32, Flow cytometry (1:100)	BD Biosciences	Cat. No. 553141; RRID:AB_394656
BV786 Rat Anti-Mouse CD45 (30-F11), Flow cytometry (1:200)	BD Biosciences	Cat. No. 564225; RRID:AB_2716861
APC-Cy™7 Rat Anti-CD11b, Flow cytometry (1:250)	BD Biosciences	Cat. No. 561039; RRID:AB_396772
BB700 Hamster Anti-Mouse CD11C, Flow cytometry (1:250)	BD Biosciences	Cat. No. 566505; RRID:AB_2869773
BV786 Mouse Anti-Human CD3, Flow cytometry (1:50)	BD Biosciences	Cat. No. 566781; RRID:AB_2869863
BUV395 Mouse Anti-Human CD4, Flow cytometry (1:50)	BD Biosciences	Cat. No. 563552; RRID:AB_2738273
BUV805 Mouse Anti-Human CD8, Flow cytometry (1:50)	BD Biosciences	Cat. No. 612889; RRID:AB_2833078
APC Mouse Anti-Human CD25, Flow cytometry (1:50)	BD Biosciences	Cat. No. 340939; RRID:AB_400551
Anti-Ki67 antibody, Immunofluorescence (1:250), 1:100 for 3D	Abcam	Cat. No. ab15580; RRID:AB_443209
Cardiac Troponin T (cTnT) Mouse Monoclonal Antibody (13-11), Immunofluorescence (1:500), 1:250 for 3D	ThermoFisher Scientific	Cat. No. MA5-12960; RRID:AB_11000742
Ki-67 Antibody, anti-human/mouse, Vio® R667, Immunofluorescence (1:50-1:100), 1:50 for 3D	Miltenyi Biotec	Cat. No. 130-120-422; RRID:AB_2801762
Anti-Actin, α-Smooth Muscle-Cy3™ antibody, Mouse monoclonal, Immunofluorescence (1:250)	Sigma Aldrich	Cat. No. C6198; RRID:AB_476856
β-Catenin Antibody, anti-human/mouse, PE, Immunofluorescence (1:100)	Miltenyi Biotec	Cat. No. 130-122-006; RRID:AB_2784495
Alexa Fluor® 647 Mouse Anti-Cardiac Troponin T (cTnT), Immunofluorescence (1:250),1:100 for 3D	BD Biosciences	Cat. No. 565744; RRID:AB_ 2739341
PE Mouse Anti-Cardiac Troponin T (cTnT), Immunofluorescence (1:250)	BD Biosciences	Cat. No. 564767; RRID:AB_2738939
Alexa Fluor® 647 anti-human/mouse/rat PCNA Antibody, Mouse IgG2a, κ, PC10 Immunofluorescence (1:250)	Biolegend	Cat. No. 307912; RRID:AB_2267947
CD31 (PECAM-1) Monoclonal Antibody (390), PE-Cyanine7, Flow cytometry (1:250)	ThermoFisher Scientific	Cat. No. 25-0311-82; RRID:AB_2716949
PE Rat Anti-Mouse CD140A, Clone APA5 Flow cytometry (1:200)	BD Biosciences	Cat. No. 562776; RRID:AB_2737787
PE anti-mouse CD140a Antibody, Flow cytometry (1:200)	Biolegend	Cat. No. 135905; RRID:AB_1953268
Nkx-2.5 Antibody (H-114), rabbit polyclonal IgG, Immunofluorescence (1:200)	Santa Cruz	Cat. No. sc-14033; RRID:AB_650281
COL1A1 (E8F4L) XP® Rabbit mAb (Alexa Fluor® 647 Conjugate), Immunofluorescence (1:100)	Cell Signaling Technology	Cat. No. 72827; RRID:AB_3696808
E-Cadherin (24E10) Rabbit mAb, Immunofluorescence (1:250)	Cell Signaling Technology	Cat. No. 3195T; RRID:AB_2291471
MLC2v Antibody, anti-human/mouse/rat, PE, Immunofluorescence (1:100)	Miltenyi Biotec	Cat. No. 130-119-680; RRID:AB_2751799
MLC2a Antibody, anti-human/mouse/rat, APC, Immunofluorescence (1:100)	Miltenyi Biotec	Cat. No. 130-118-674; RRID:AB_2751549
Various Alexa Secondary Antibodies Immunofluorescence (1:500), 1:250 for 3D	ThermoFisher Scientific	N/A

**Biological samples**		

Healthy human donors: Na Heparin anti-coagulated blood	St Vincent’s Hospital	N/A

**Chemicals, peptides, and recombinant proteins**		

Flash Phalloidin™ Red 594, Staining (1:100)	Biolegend	Cat. No. 424203
Bodipy BDP® 630/650 lipid stain, Immunofluorescence (250 nM)	Lumiprobe	Cat. No. 1233-1 mg
0.4% tricaine (MS 222)	Sigma-Aldrich	Cat. No. E10521
N,N,N′,N′-Tetrakis(2-hydroxyethyl)ethylenediamine, THEED	Sigma-Aldrich	Cat. No. 87600; Cas No. 140-07-8
Recombinant human PDGF-BB	PeproTech Inc.	Cat. No. 100-14B
Recombinant human FGF-2	Miltenyi Biotec	Cat. No. 130-093-837
AZDye 488 Picolyl Azide	Vector Laboratories	Catalog No. CCT-1276
AZDye 555 Picolyl Azide	Vector Laboratories	Catalog No. CCT-1288
AZDye 633 Picolyl Azide	Vector Laboratories	Catalog No. CCT-1549
AZDye 680 Picolyl Azide	Vector Laboratories	Catalog No. CCT-1511
Lithium borohydride (LiBH_4_)	Sigma-Aldrich	Cat. No. 222356; Cas No. 16949-15-8
5-Ethynyl-2′-deoxyuridine (EdU)	MedChem Express	Cat. No. HY-118411
Aphidicolin	MedChemExpress	Cat. No. HY-N6733
Ethyl cinnamate	Sigma-Alrich	Cat. No. 112372; CAS No.: 103-36-6
DMSO Hybri-Max	Sigma-Aldrich	Cat. No. D2650

**Critical commercial assays**		

Click-iT Plus EdU Alexa Fluor 488 Flow Cytometry Assay Kit	Invitrogen by Thermo Fisher Scientific	Cat. No. C10632; LOT 2291430
Click-&-Go Plus EdU 488 Flow Cytometry Kit	Click Chemistry Tools	Cat. No. 1375
Click-iT™ EdU Cell Proliferation Kit for Imaging with Alexa Fluor™ 488 dye	Invitrogen by Thermo Fisher Scientific	Cat. No. C10337

**Experimental models: Cell lines**		

Mouse: C3H/10T1/2, Clone 8 cells, passage 14	ATCC (CCL-226)	RRID:CVCL_0190
Mouse: C2C12 myoblasts, passage 12	ATCC (CRL-1772)	RRID:CVCL_0188
Human: Passage 35 hiPSCs-FUCCI	MCRIi010-A	RRID:CVCL_UK91
Human: hiPSCs	HPSI0314i-hoik_1	RRID:CVCL_AE82
Human: hiPSCs	CMRIi0013-A-6-9CMRI	Gifted by Dr Anai Gonzalez-Cordero at Children’s Medical Research Institute
Human: passage 60 MCF7	ATCC (HTB-22)	RRID:CVCL_0031
Human: passage 25 MDA-MB-231	ATCC (HTB-26)	RRID:CVCL_0062
Human: passage 16 MIA PaCa-2 cells	ATCC (CRL-1420)	RRID:CVCL_0428
Human: passage 20 Panc-1	ATCC (CRL-1469)	RRID:CVCL_0480

**Experimental models: Organisms/strains**		

Wild-type zebrafish (Danio rerio); Ekkwill (EK) strain	Dr Kazu Kikuchi Lab	Gifted by Dr Emily Wong at Victor Chang Cardiac Research Institute
Wild type [WT, Inbred C57BL/6J]	Jackson Laboratory	000664

**Software and algorithms**		

BD FACSDiva™ Software	BD Biosciences	RRID:SCR_001456
ZEISS ZEN blue 3.4 for LSM900	Zeiss	RRID:SCR_013672
ZEISS ZEN Black software for Z.1	Zeiss	RRID:SCR_018163
Fiji software (ImageJ2, version: 2.14.0/1.54f)	Schindelin et al. 2012^76^	RRID:SCR_002285

### Experimental model and study participant details

#### Ethics statement and mouse strain

Wild type [WT, Inbred C57BL/6J] (Jackson Laboratory; 000664) mice were bred and housed under pathogen-free conditions in the BioCORE facility (Victor Chang Cardiac Research Institute, Sydney, Australia). All experimental procedures were approved by the Garvan Institute/St. Vincent’s Hospital Animal Experimentation Ethics Committee (No. 19/07, 19/14), and performed in strict accordance with the National Health and Medical Research Council (NHMRC) of Australia Guidelines on Animal Experimentation. Mice were maintained on a 12-h light/dark cycle from 6 a.m. to 6 p.m. and had unrestricted access to food and water. All efforts were made to minimize animal suffering. We used 3-6-month-old WT male and female animals for our EdU studies.

#### Subjects and ethics

Recruitment of healthy adult donors through St Vincent’s Hospital was approved by St Vincent’s Hospital Human Research Ethics Committee (HREC/13/SVH/145 and HREC/10/SVH/130). All participants gave written informed consent.

#### Zebrafish maintenance and breeding

Wild type *Danio rerio* zebrafish (Ekkwill (EK) strain) were maintained at the Victor Chang Cardiac Research Institute. All procedures were approved by the Garvan Institute of Medical Research/St Vincent’s Hospital Animal Ethics Committee under Animal Research Authorities (AEC:22_25). Adult zebrafish were housed in 3.5 L tanks Z-Hub system (Aquatic Habitats), with a maximum of 30 fish per tank. They were kept in recirculating chlorine-free water at 27 ± 1 °C, exposed to a 14.5:9.5-h light-dark cycle, and fed twice daily. Fertilized embryos were incubated at 28°C (Memmert, Büchenbach, Germany) in Embryo Media, which consisted of 0.03% (w/v) ocean salt (Aquasonic, Wauchope, Australia), 0.0075% (w/v) calcium sulfate, and 0.00002% (w/v) methylene blue in water, at a density of 60–100 embryos per 25 mL dish. At 5 days post-fertilization (dpf), viable free-swimming zebrafish larvae were visually screened. For adult zebrafish EdU experiments, adult fish were intraperitoneally injected with 50 μL of 8 mM EdU once daily at 5-, 6-, and 7-day post-injury. Sex determination and segregation of zebrafish was not performed in the embryonic stage (48–120 h postfertilization).

### Method details

#### PubMed search

To determine the number of PubMed articles mentioning bromodeoxyuridine (BrdU) and ethynyl deoxyuridine (EdU), a public search was conducted on 13 January 2025 using the PubMed database (https://pubmed.ncbi.nlm.nih.gov/). Search results per year were downloaded as CSV files and subsequently plotted for analysis.

#### Reagents

Recombinant human (rh) PDGF-BB (100-14B, PeproTech Inc., US) was reconstituted in PBS1X containing 0.01% BSA (Sigma-Aldrich, 05470), according to the supplier’s instructions, and used as indicated in the corresponding figures. Lyophilized rhFGF-2, research-grade (130-093-837, Miltenyi Biotec) was reconstituted with deionized sterile-filtered water. 5-Ethynyl-2′-deoxyuridine (EdU) (MedChem Express, HY-118411, 100 mg) was reconstituted in DMSO Hybri-Max (Sigma-Aldrich, D2650). Other reagents, unless otherwise indicated, were purchased from Sigma-Aldrich.

#### Cell culture and cell growth of C3H/10 T1/2 and C2C12 cells

The murine mesenchymal stromal cell line C3H/10T1/2, Clone 8 (CCL-226, ATCC, VA, USA) and C2C12 myoblast cell line (CRL-1772, ATCC) were grown at 37°C in 5% CO_2_ and cultured in DMEM, high glucose, GlutaMAX™ Supplement (Cat. No. 10566016), supplemented with 10% (v/v) heat-inactivated fetal bovine serum (FBS; Hyclone, UT, United States) and antibiotics (Penicillin-Streptomycin, Cat. No. 15140122, Gibco by Life Technologies). Cells were passaged every other day and used between passages 10–20. Additionally, cells were treated with rhPDGF-BB and rhFGF-2 in DMEM supplemented with 1% FBS and antibiotics at 37°C in 5% CO_2_, at concentrations and times indicated in the corresponding figure legend. Our cell cultures were periodically tested for mycoplasma contamination using PCR at the Garvan Institute of Medical Research.

#### hiPSC cell culture

The male XY hiPSC line PB010.5 (MCRIi010-A (RRID:CVCL_UK91)) with the incorporation of a reporter YFPhGEMININ/mCherryhCDT1 FUCCI cassette into the EEF2 locus, which exhibited no karyotypic abnormalities, was generated by the iPSC Derivation & Gene Editing Facility (Murdoch Children’s Research Institute, Melbourne, Australia). These hiPSCs were split using ReLeSR™ (100–0483, STEMCELL Technologies) and maintained in mTeSR™ Plus (100–0276, STEMCELL Technologies) with 0.5% v/v Penicillin-Streptomycin (15140122, Gibco) under feeder-free conditions on hESC pre-screened Matrigel (354277, Corning). hiPSCs were used between passages 30 to 45. To ensure the quality and pluripotency status of hiPSCs over long-term maintenance, we periodically conducted standard flow cytometric immunophenotyping (Miltenyi Biotec-tested panel 25, Miltenyi Biotec), as well as assessed the growth rate and morphology of hiPSC colonies. hiPSCs were periodically tested for mycoplasma contamination using PCR at the Garvan Institute of Medical Research.

#### Cancer cell growth protocol and conditions

Cancer cell lines were grown at 37°C in 5% CO_2_ and cultured in DMEM, high glucose, GlutaMAX™ Supplement (Cat. No. 10566016), supplemented with 10% (v/v) heat-inactivated fetal bovine serum (FBS; Hyclone, UT, United States) and antibiotics (Penicillin-Streptomycin, Cat. No. 15140122, Gibco by Life Technologies). Panc-1 cells (passage 20) were cultured at a density of 16,000 cells/cm^2^, MCF7 cells (passage 60) at 25,000 cells/cm^2^, MDA-MB-231 cells (passage 25) at 12,000 cells/cm^2^, and MIA PaCa-2 cells (passage 16) at 16,000 cells/cm^2^. Cells were pre-plated onto 6-well plastic tissue culture dishes and incubated at 37°C in a 5% CO_2_ atmosphere for about 24 or 48 h. Before the EdU thymidine analog treatment, the growth medium was replaced with a fresh medium for 2 h. Subsequently, 10 μM of EdU (MedChem Express, HY-118411) was added to the cultures as indicated in the figure legends and text.

#### Fixation protocol for flow cytometry: Cultured cell lines and hiPSCs

For fixing cell lines (C2C12, NIH3T3, C3H-10T1/2, cancer cell lines) and hiPSCs for flow cytometry, cells in 6-well plates were processed as follows. The media was removed, and each well was washed once with 1 mL of PBS. Then, 1 mL of TrypLE was added to each well, and the plates were incubated for 5–7 min at 37°C. The cells were pipetted up and down a few times per well to obtain a single-cell suspension, which was corroborated using a microscope. Next, 1 mL of cold FACS Buffer (PBS 1×, 2% FBS v/v, 2 mM EDTA pH 7.9) was added to each well to inactivate the TrypLE. The cells from each well were transferred to separate 2 mL tubes and spun at 500g for 5 min (200g for 3 min for hiPSCs). The supernatant was removed, and the pellets were resuspended by flicking the tubes. Then, 1 mL of FACS Buffer was added to each well. The tubes were checked for clumps by looking at them against a light source, and any clumps present were removed by flicking the tubes. 4% PFA was added to a final concentration of 2% in each tube, and the cells were fixed for 10 min at room temperature. After fixation, the cells were washed with abundant PBS, and the tubes were spun at 400g for 4 min (200g for 3 min for hiPSCs). The supernatant was removed, and the pellets were resuspended by flicking. Finally, 1–2 mL of PBS with 0.02% Sodium Azide was added to each tube, and the samples were stored at 4°C. The volumes were adapted when using smaller well plates.

#### Cytospin of cells in suspension

After mounting CytoSep™ Cytology Funnels (base holder and fluid chamber) for the Sakura Cyto-Tek® Cytocentrifuge (Model 4323, Sakura) and a white filter for the Sakura cytology funnel (M963FW, Simport), PFA-fixed cells in suspension were centrifuged (2,000 rpm, 3 min) onto Epredia™ SuperFrost Plus™ Adhesion slides (12312148, Fisher Scientific). Following the creation of a hydrophobic barrier with a PAP pen (ab2601, Abcam), the cells were ready for staining. We typically used 0.1 to 0.8 mL of cells in suspension.

#### OpenEMMU click chemistry for flow cytometry combined with antibody staining

For flow cytometric analysis of cells, the required number of fixed cells was transferred into 1.5 mL Eppendorf tubes. We typically used between 100,000 and 1,000,000 cells. Each tube received 1 mL of 1% BSA (BSAS-AU 100g, Bovogen Biologicals) in PBS, followed by centrifugation at 500*g* for 5 min. The supernatants were discarded, and the pellets were resuspended by flicking. Subsequently, 500 μL of 1X in-house permeabilization/wash buffer^77^ (0.2% (w/v) saponin containing either 4% FBS (v/v) or 2% NCS (v/v), 1% (w/v) BSA and 0.02% (v/v) Sodium Azide in PBS) was added to each tube, and the samples were incubated at room temperature (RT) for 10 min. After incubation, the tubes were centrifuged again at 500g for 5 min. The supernatants were discarded, leaving 50 μL in each tube, and the pellets were resuspended by flicking. The EdU mix was then prepared by sequentially adding the following components: 909 μL PBS, 80 μL of Copper(II) sulfate pentahydrate (209198, Sigma Aldrich; 10 mM fresh or frozen stock, final concentration 800 μM), 1 μL Fluor-Azide (200 μM stock, final concentration 200 nM), and 10 μL freshly made L-Ascorbic acid (100 mg/mL frozen stock, final concentration 5.7 mM). 300–450 μL of the EdU mix was added to each tube, and the samples were incubated at RT for 30 min, covered from light. Following this, the tubes were washed twice with 1 mL of 1% BSA in PBS and once with 0.5 mL of 1X permeabilization/wash buffer, and centrifuged at 500g for 5 min in between washes. The supernatants were discarded, leaving 50 μL in each tube, and the pellets were resuspended by flicking. 100 μL of primary or conjugated antibodies, prepared in 1X permeabilization/wash buffer, was added to the relevant tubes, and the samples were incubated overnight at 4°C. The next day, the tubes were washed once with 1 mL of 1% BSA in PBS and once with 0.5 mL of 1X perm/wash buffer, followed by centrifugation at 500*g* for 5 min. The supernatants were discarded, leaving 50 μL in each tube, and the pellets were resuspended by flicking. When using secondary antibodies, 50 μL of them, prepared in 1X permeabilization/wash buffer, was added to the relevant tubes, and the samples were incubated at RT for 60–120 min, covered. After this incubation, the tubes were washed once with 1 mL of 1% BSA in PBS and once with 0.5 mL of 1X perm/wash buffer. The supernatants were discarded, leaving approximately 100 μL in each tube, and the pellets were resuspended by flicking. A 1:2500 dilution of Vybrant™ DyeCycle™ Violet (V35003, 5 mM) in 1X perm/wash buffer was prepared, and 100 μL was added to each tube for a final concentration of 1:5000. The tubes were covered and taken for analysis by flow cytometry. Details about the AZDye fluorescent probes and antibodies used in this study can be found in the Supplementary Materials (key resources table). L-Ascorbic acid, Copper(II) sulfate pentahydrate, and Picolyl Azide, all fluorescent probes, were aliquoted and stored at −20°C for regular use.

#### In-house OpenEMMU click chemistry for immunofluorescence and antibody staining

Cells, either in a cytospin slide and/or PhenoPlate 96-well, black, optically clear flat-bottom plates (Revvity), were washed with 1X PBS and then permeabilized in 1× saponin-based permeabilization/wash buffer for 15 min. During this incubation period, the EdU mix was prepared. After 15 min, the 1X permeabilization/wash buffer was removed, and the samples were incubated with the EdU mix (same formula as for flow cytometry) at RT for 30 min, covered from the light. Following this, the samples were washed three times with 1X PBS. The samples were then incubated overnight at 4°C with primary or conjugated antibodies prepared in 1X permeabilization/wash buffer. The next day, the samples were washed three times with 1X PBS before adding secondary antibodies, also prepared in 1X permeabilization/wash buffer, and incubated at RT for 60–120 min, covered. After this incubation, the samples were washed three times with 1X PBS. Finally, a 1:5000 dilution of Vybrant™ DyeCycle™ Violet in 1X permeabilization/wash buffer was prepared, and the samples were incubated with this solution and stored at 4°C until they were ready for imaging. Details about the AZDye fluorescent probes and antibodies used in this study can be found in the Supplementary Materials (key resources table).

#### Direct comparison of OpenEMMU and Click-iT flow cytometry kit and EdU detection

Direct flow cytometry-based comparisons were performed using hiPSCs, hiPSC-derived cardiomyocytes (cTnT^+^ fraction), bone marrow cells, C2C12 myoblasts and spleenocytes. OpenEMMU was applied as described above for flow cytometry assay, while the Click-iT™ EdU Alexa Fluor™ 488 Flow Cytometry Assay Kit (Catalog number C10425) was used according to the manufacturer’s instructions. For each comparison, samples (*n* = 3) were divided equally to ensure identical cell populations were analyzed with both methods.

#### Direct comparison of OpenEMMU and Click-iT imaging kit for immunofluorescence and EdU detection

Direct imaging-based comparisons were performed using hiPSCs seeded in PhenoPlate 96-well plates. OpenEMMU was applied as described above for imaging, while the Click-iT™ EdU Cell Proliferation Kit for Imaging with Alexa Fluor™ 488 dye (Catalog number C10337) was used according to the manufacturer’s instructions. For each comparison, samples (*n* = 3) were imaged using identical acquisition parameters (laser power and gain settings) to ensure consistent detection sensitivity and fluorescence intensity across both methods.

#### hiPSC-derived cardiomyocyte in-house differentiation protocol

On Day −2, hiPSCs were dissociated using pre-warmed TrypLE (TrypLE Express Enzyme (1×), no phenol red, Cat. No. 12604013, ThermoFisher Scientific, USA) for 7 min and seeded at a density of 200,000 cells/cm^2^ in 24 or 48-well plates using mTeSR medium supplemented with 5 μM Y-27632. On Day −1, the media in the wells was replaced with fresh mTeSR. On Day 0, when the wells were 70–90% confluent, they were washed once with PBS and 6 μM CHIR99021 in RPMI+B27(-ins)+Glutamax was added. Approximately 24 h later, on Day 1, the wells were washed once with PBS, and RPMI+B27(-ins)+Glutamax with 1 μM CHIR99021 was added. On Day 3/4, the wells were washed once with PBS and RPMI+B27(-ins)+Glutamax supplemented with 200 μM L-Ascorbic acid, 5 μM IWP-2, and 5 μM XAV939 was added. 48 h after Day 3/4, on Day 5/6, the wells were washed once with PBS and RPMI+B27(-ins)+Glutamax supplemented with 200 μM L-Ascorbic acid added. On Day 7, the media was replaced with RPMI+B27+Glutamax supplemented with 200 μM L-Ascorbic acid. From Day 9 onwards, the media was replaced every 2–3 days with RPMI+B27+Glutamax supplemented with 200 μM L-Ascorbic acid.

#### hiPSC-derived cardiomyocyte in-house dissociation protocol

Cells in 48-well plates were washed with 0.5 mL/well PBS and then incubated with 300 μL/well of room temperature custom Cardiomyocyte Dissociation Buffer for 30 min at 37°C, tapping the plate every 15 min. The Cardiomyocyte Dissociation Buffer was composed of 12.5 U/mL Papain (Cat. #10108014001, Sigma Aldrich), 0.5 mM Cysteine, 132.5 U/mL Collagenase II (Cat. #LS004176, Worthington), 0.5% BSA (Cat. #A7906, Sigma), 0.025 mg/mL DNase I (Cat. #10104159001, Sigma Aldrich), and 0.1 U/mL Dispase (Cat. #07913, STEMCELL), all made up in DMEM (+L-Glutamine, +Sodium Pyruvate, +4.5 g/L Glucose). Following this, 500 μL FACS buffer/well was added, and the plate was placed on ice while still in the hood. Cells were gently pipetted up and down 20 times using a P1000 pipette to achieve single-cell status, with an additional 10 pipetting cycles if needed. The cells were then transferred to 15 mL Falcon tubes, pooling wells as required, and excess FACS buffer was added to each tube. The tubes were spun at 300g for 4 min, and the supernatant was carefully removed. The cell pellets were resuspended by flicking. Cells were fixed in 2% PFA (prepared by diluting 4% PFA stock in FACS buffer) for 10 min. After fixation, excess PBS was added to the tubes, and they were spun down at 300g for 4 min. The supernatant was removed, and the pellets were resuspended, ensuring no clumps were present. Finally, sufficient PBS (+0.02% Sodium Azide) was added to each tube, and the cells were stored at 4°C.

#### Mouse dissection for the spleen, bone marrow, and small intestine

Mice were asphyxiated in a CO_2_ chamber at a slow fill rate (approximately 20–30% of the chamber volume per minute). Death was confirmed by cervical dislocation and a lack of response to physical contact. After immobilizing the deceased mouse, the chest fur was lightly sprayed with 80% ethanol. The chest cavity was then opened using external dissection forceps and scissors. The spleen and small intestine were removed and transferred to a dish containing ice-cold DMEM (Cat. No. 10566016) for washing. For the small intestine, a 2 cm section of the ileum was cut, and the digested food was removed by flushing the intestine twice with 10 mL of cold PBS1x. The ileum was fixed in 4% PFA for 30 min at room temperature, washed with abundant PBS1x, and stored in 100% methanol at −20°C until further FFPE or tissue-clearing processing. The proportion of EdU-positive, Ki67-negative (EdU+Ki67-) cells in z stack images was quantified manually (blinded) using the *Cell Counter* plugin in Fiji (*n* = 7). This was performed on individual and merged channels.

#### Splenocytes isolation, fixation and staining

After EdU uptake, spleens were individually washed in cold DMEM and placed in a 100 μm cell strainer, then carefully ground until forming a paste-like slurry. Five milliliters of cold FACS buffer were used to wash the slurry as it passed through the strainer. The remaining tiny pieces of spleen were further ground, followed by another 10 mL of cold FACS buffer. The mixture was centrifuged at 400 g for 5 min at 4°C, and the supernatant was discarded. The pellet was resuspended by flicking the tube for about 5–7 s to ensure complete resuspension. Five milliliters of ACK/RBC lysis buffer was added to the resuspended pellet and incubated for 3 min on ice. Ten milliliters of cold FACS buffer were then added to stop the lysis, followed by centrifugation at 400*g* for 5 min at 4°C, and the supernatant was discarded. The pellet was resuspended by flicking the tube and 5 mL of cold FACS buffer was added. The splenocytes were filtered through a blue cap flow cytometry tube (40 μm) and then fixed in 1% PFA for 10 min at room temperature. The cells were washed with FACS buffer and centrifuged at 500 g for 5 min, and the supernatant was carefully discarded. The cells were resuspended in 5 mL of 1% BSA in PBS1x and 0.02% (v/v) Sodium Azide. OpenEMMU click chemistry was then performed using 500,000 to 1,000,000 total cells according to the OpenEMMU protocol for flow cytometry. After Fc blocking (anti-Mouse CD16/CD32 antibody), antibodies were prepared in a 1X in-house permeabilization/wash buffer and incubated with cells for 1 h at room temperature. Details about the AZDye fluorescent Picolyl Azide and antibodies used in this study can be found in the key resources table.

#### Bone marrow cell isolation, fixation and staining

After EdU uptake, the tibia and femur bones of adult WT mice (both male and female) were separated from the surrounding muscular and connective tissue. Both ends of the isolated long bones were cut using a surgical blade, and the bone marrow cells were flushed out with FACS buffer using an Insulin Syringe (29G x 8 mm). The cells were collected in 2 mL Eppendorf tubes, filtered through a pluriStrainer Mini 40 μm (43-10040-50, pluriSelect) into a new tube, and then fixed in 1% PFA. The cells were washed with FACS buffer and centrifuged at 500 g for 5 min, and the supernatant was carefully discarded. OpenEMMU click chemistry was then performed using 500,000 to 1,000,000 total cells as described above. After Fc blocking (anti-Mouse CD16/CD32 antibody), antibodies were prepared in a 1X in-house permeabilization/wash buffer and incubated with cells for 1 h at room temperature. Details about the AZDye fluorescent Picolyl Azide and antibodies used in this study can be found in the supplemental information (key resources table).

#### Embryonic heart cell isolation and fixation

Following 3 h of embryonic EdU uptake, E14-E15 wild-type (WT) embryonic hearts from both male and female mice were isolated and cleaned of surrounding tissue. The hearts were then cleared of blood using cold PBS. Each heart was dissociated into single cells with the *Cardiomyocyte Dissociation Buffer* (0.5–0.75 mL buffer per tube) with agitation at 900 RPM for 30–40 min at 37°C in 2 mL Eppendorf tubes. Following this, 1 mL FACS buffer/tube was added, and the dissociated cells were filtered through a pluriStrainer Mini 100 μm (43-10100-50, pluriSelect) into a new 2 mL tube, and fixed in 1% PFA for 10 min. The cells were washed with FACS buffer and centrifuged at 500 g for 5 min, and the supernatant was carefully discarded. OpenEMMU click chemistry and antibody staining was subsequently performed on 100,000 to 500,000 total cells as previously described.

#### Multi-parametric flow cytometry analyses and fluorescence-activated cell sorting (FACS)

Multi-parametric flow cytometry analyses were performed using a BD LSRFortessaTM X-20 Cell Analyzer and a BD FACSymphony A5 High-Parameter Cell Analyzer, both equipped with five excitation lasers (UV 355 nm, Violet 405 nm, Blue 488 nm, Yellow/Green 561 nm, and Red 633 nm). FSC-H versus FSC-A, FSC-H versus FSC-W, and SSC-H versus SSC-W cytograms were used to discriminate and gate out doublets/cell aggregates during sorting or analysis. After a 1-h EdU labeling and fixation with 2% paraformaldehyde (PFA), a BD FACSAria™ III Cell Sorter (70 μm nozzle) was used to sort the hiPSCs. Cells were first gated for singlets, followed by bivariate analysis based on DNA content and EdU incorporation. Data were collected using BD FACSDiva™ Software. For optimal DNA dye signal detection and cell cycle progression analyses, an event concentration of <1,000 events/seconds was used, and 10,000–50,000 events were captured for most cell types. For analysis of complex organs and tissues, including the spleen and bone marrow, around 500,000–1,000,000 events were captured. All flow cytometry data were analyzed using FlowJo Portal (version 10.8.1, BD) using macOS Monterey. Automated or manual compensation was done only when exclusively required.

#### Confocal laser scanning microscopy

Confocal laser scanning microscopy of cells and tissues was performed using a Zeiss LSM900 inverted confocal laser scanning microscope, which includes an upright Zeiss Axio Observer 7, Colibri 5 solid-state LED fluorescence light sources (solid-state laser lines: 405, 488, 561, 640), two Gallium Arsenide Phosphide photomultiplier tubes (GaAsP-PMT), and a motorized stage controlled by ZEN blue 3.4 software. Regular or tile images were acquired using objectives ranging from ×10 (0.45 NA with a WD 2.0, Air Plan-APO UV-VIS–NIR), ×20 (0.8 NA with a WD 0.55, Air Plan-APO UV-VIS–NIR), ×40 (1.3 NA with a WD 0.21, Oil Plan-APO DIC-UV-VIS–IR), and ×63 (1.2 NA with a WD 0.19, Oil Plan-APO DIC-UV-VIS–IR). The ×40 and ×63 objectives were used with immersion oil ImmersolTM 518 F (433802-9010-000, Zeiss). For z stack 3D imaging, z-step sizes ranged from 0.25–1 μm, with images acquired under confocal settings using a motorized focus drive. Laser power during imaging was kept below 3.5%. When indicated, some tissue FFPE sections were imaged using a Leica Thunder Imager and Leica Image Files (LIFs) were imported directly in Fiji.

#### Timed mating and dissection of the mouse embryo for FFPE

Timed mating was performed between wild-type (WT) male and female mice, with pregnancy confirmed by the detection of a copulation plug. Pregnant females received an intraperitoneal injection of 20 mg/kg EdU (using a 100 mM EdU stock) in sterile 0.9% NaCl solution between embryonic days E10 and E14.5. Three hours post-injection, the pregnant mice were sacrificed as previously described, and embryos were collected in plastic Petri dishes containing ice-cold PBS1x. The embryos were briefly rinsed in ice-cold PBS1x to remove any residual blood, then immersed in 4% PFA and placed on a roller for 3 h at room temperature. Embryonic hearts were isolated by surgical removal. The PFA solution was then replaced with PBS1x, and whole embryos or isolated hearts were stored in 100% methanol at −20°C until further FFPE processing.

#### Formalin-fixed paraffin-embedded (FFPE) tissue processing and embedding

All tissues, organs, embryos, and specimens designated for FFPE were initially fixed in 4% paraformaldehyde (PFA) and subsequently preserved in 100% methanol for later processing. Tissue samples were then processed using the Leica Peloris 3, following one of the standard protocols, which lasted either 4 or 8 h and involved the same steps and reagents. The samples progressed through 70% ethanol, 90% ethanol, and 100% ethanol, followed by Xylene at room temperature, and finally paraffin at 60°C. The protocol was chosen based on the sample type and size. Generally, small samples (less than 3 mm small/thin) were processed on the 4-h run. Samples were then embedded using the Leica HistoCore Arcadia H&C.

#### Sectioning, H&E, and IHC

Blocks were sectioned at 5 μm using a Leica RM2235 microtome. Sections were placed on either plain glass slides or positively charged slides and incubated for 2 h in a 60°C oven to ensure maximum adhesion. H&E is a regressive stain, and the standard H&E procedures were followed using the Leica ST5010 Autostainer XL with Hematoxylin (Hematoxylin Harris non-toxic (acidified); Australian Biostain) and Eosin (Eosin Phloxine Alcoholic 1%; Australian Biostain).

#### Deparaffinization and antigen retrieval protocol for FFPE tissues

We used an optimized protocol for FFPE, deparaffinization, and antigen retrieval.^45^ In brief, FFPE slides were incubated in Xylene for 15 min, repeated three times. This was followed by incubation in a 1:1 mixture of Xylene and ethanol for 5 min. Slides were then incubated in 100% ethanol for 3 min, repeated twice, followed by 95% ethanol for 3 min, repeated twice, and 70% ethanol for 3 min, repeated twice. Finally, slides were washed in PBS for 3 min, and repeated twice. For antigen retrieval, Citrate Buffer was prepared by dissolving 2.94 g of Trisodium Citrate Dihydrate in 1000 mL of distilled water. The pH was adjusted to 6 using 1M HCl, and 0.5 mL of Tween 20 was added. The Citrate Buffer was heated on a hotplate, and when the temperature reached 95°C, slides were placed in the beaker and incubated for 15 min. After incubation, slides were removed from the buffer and allowed to cool at room temperature for 10 min. The slides were then ready to be stained.

#### Ethyl cinnamate tissue clearing and click labeling of mice intestines

Tissues stored in 100% methanol at −20°C were rehydrated through successive 5-min incubations in 75%, 50%, and 25% methanol prepared in PBSTx (PBS +0.3% Triton X-100). This was followed by two washes in PBSTx for 5 min each, and then two washes in PBS for 5 min each. The tissues were de-pigmented in 3% H_2_O_2_ (prepared in 0.8% KOH) for 5 min at room temperature. After de-pigmentation, the tissues were washed four times in PBS. They were then permeabilized in PBSTx for 60 min at room temperature and subsequently overnight at 4°C. The next day, most of the supernatant was removed, and 500 μL of fresh OpenEMMU reaction mix (prepared in PBSTx or Saponin-based perm/wash buffer) was added to each tube using AZDye 488. The tubes were incubated and covered at room temperature for 60 min. Following this, the tissues were washed four times in PBS. Five hundred microliters of a solution containing conjugated antibodies or CF®640R WGA (29026, Biotium) (prepared in Zebrafish permeabilization/wash buffer: 0.4% Saponin, 4% FBS, 1% BSA, 10% DMSO, 1% Triton X-100, 0.02% Sodium Azide in PBS) was added to each tube and incubated covered at room temperature for 30 min. The tissues were then washed four times in PBS. The tubes were dehydrated through successive 30-min incubations at room temperature with rocking in 30%, 50%, and 70% propane-2-ol (prepared in PBS), followed by 100% propane-2-ol. The tubes were then dehydrated overnight in 100% propan-2-ol at 4°C. The next day, propan-2-ol was replaced with ethyl cinnamate (112372, Sigma-Alrich), and the tubes were incubated and covered on a rocker. The ethyl cinnamate was replaced after 1 h to remove any traces of propan-2-ol. The tubes were then incubated at room temperature on a rocker overnight. Finally, the intestines were imaged while still in ethyl cinnamate using laser confocal imaging.

#### Deep clearing and click labeling of mice embryos

Embryos stored in methanol were rehydrated through consecutive 10-min incubations in 75%, 50%, and 25% methanol in PBSTx0.3 (0.3% Triton X-100 in PBS). This was followed by two washes in PBSTx0.3. The embryos were then permeabilized in ice-cold acetone overnight at −20°C. After permeabilization, embryos were washed four times in PBS for 5 min each. Embryos were transferred into glass vials and 5 mL of Solution-1 (25% Urea, 9% THEED, 5% Triton X-100 in water) was added. The vials were incubated on a rocking platform with gentle rocking at 37°C for 5 days, with fresh Solution-1 added every 2–3 days or whenever the solution appeared yellowish. Following this, embryos were washed three times with 4 mL of PBS for 10 min each. Blocking was performed by adding 4 mL of blocking PBSTx (4% FBS, 1% Triton X-100, 1% BSA in PBS) to each vial and incubating on a rocking platform with gentle rocking at 37°C overnight. Embryos were then transferred into 2 mL Eppendorf tubes and washed twice in PBSTx0.3 for 10 min each. An EdU mix was prepared using PBSTx1 (1% Triton X-100 in PBS) and 1 mL was added to each tube. The embryos were incubated on a rocking platform with gentle rocking at 37°C overnight. Following EdU click chemistry incubation, tubes were washed four times in PBSTx0.3 for 15 min each on a rocking platform with gentle rocking at 37°C. Primary antibodies were prepared in Zebrafish perm/wash buffer (0.4% Saponin, 4% FBS, 1% BSA, 10% DMSO, 1% Triton X-100, 0.02% Sodium Azide in PBS). 500 μL of the antibody mix was added to each tube and incubated on a rocking platform with gentle rocking at 37°C for 7 days. This was followed by four washes in PBSTx0.3 for 15 min each on a rocking platform with gentle rocking at 37°C. Secondary antibodies, including Vybrant Violet, were prepared in Zebrafish perm/wash buffer. 500 μL of the secondary antibody mix was added to each tube and incubated on a rocking platform with gentle rocking at 37°C for 7 days. The tubes were then washed four times in PBS for 1 h each and then overnight on a rocking platform with gentle rocking at 37°C. Embryos were RI matched by incubating them in a solution of 7M Urea in PBS on a rocking platform with gentle rocking at 37°C for 5 days. Finally, the objects were analyzed using Lightsheet Microscopy.

#### Deep clearing and OpenEMMU labeling of mouse embryonic hearts

Hearts stored in methanol were rehydrated through consecutive 5-min incubations in 75%, 50%, and 25% methanol in PBSTx0.3 (0.3% Triton X-100 in PBS). This was followed by two washes in PBSTx0.3 for 5 min each. The hearts were then permeabilized in ice-cold acetone overnight at −20°C. After permeabilization, the hearts were washed four times in PBS for 5 min each. The hearts were transferred into Eppendorf tubes, and 1 mL of Solution-1 (25% Urea, 9% THEED, 5% Triton X-100 in water) was added to each tube. The tubes were incubated on a rocking platform with gentle rocking at 37°C overnight. Following this, the hearts were washed three times with 1 mL of PBS per tube for 5 min each. Blocking was performed by adding 1 mL of blocking PBSTx (4% FBS, 1% Triton X-100, 1% BSA in PBS) to each tube and incubating on a rocking platform with gentle rocking at 37°C overnight. The hearts were then washed twice in PBSTx0.3 for 5 min each. OpenEMMU mix was prepared using PBSTx1 (1% Triton X-100 in PBS), and 600 μL was added to each tube. The hearts were incubated and covered on a rocking platform with gentle rocking at 37°C overnight. Following incubation, the tubes were washed four times in PBS for 5 min each, covered on a rocking platform with gentle rocking at 37°C. Primary antibodies were prepared in Zebrafish perm/wash buffer. 200 μL of the antibody mix was added to each tube and incubated covered on a rocking platform with gentle rocking at 37°C over the weekend (approximately 3 days). This was followed by three washes in PBSTx0.3 for 10 min each on a rocking platform with gentle rocking at 37°C. 500 μL of the secondary antibody mix (prepared in Zebrafish perm/wash buffer) was added to each tube and incubated covered on a rocking platform with gentle rocking at 37°C overnight. The tubes were then washed three times in PBSTx0.3 for 30 min each on a rocking platform with gentle rocking at 37°C. The hearts were RI matched by incubating them in a solution of 7M Urea in PBS, covered on a rocking platform with gentle rocking at 37°C for 30 min. Finally, the hearts were analyzed using light-sheet microscopy.

#### Deep clearing and click labeling of mice limbs

Limbs were rehydrated by incubating in successive dilutions of methanol (75%, 50%, and 25%) prepared in PBSTx0.3 (0.3% Triton X-100 in PBS) for 10 min each. This was followed by three washes in PBSTx for 5 min each. The limbs were then permeabilized in ice-cold acetone overnight at −20°C. After permeabilization, the limbs were washed four times in PBS. The limbs were incubated in Solution-1 (25% Urea, 9% THEED, 5% Triton X-100 in water) for 6 h at 37°C on a rocker. Following this, the limbs were washed four times in PBS. They were then permeabilized overnight in PBSTx1 (1% Triton X-100 in PBS) at room temperature on a rocker. Most of the PBSTx was removed, and 500 μL of the OpenEMMU reaction mix (prepared using PBSTx1) was added to each tube. The tubes were incubated at room temperature on a rocker for 120 min. After the reaction, the limbs were washed four times in PBS. The limbs were then incubated overnight in conjugated antibodies and DNA dyes prepared in Zebrafish perm/wash buffer at room temperature on a rocker. After incubation, the limbs were washed four times in PBS. Finally, the samples were RI matched by incubating them in a solution of 7M Urea in PBS at room temperature on a rocker for an appropriate amount of time and imaged using Light Sheet fluorescence microscopy.

#### Light sheet-based fluorescence microscopy (LSFM) for imaging of large specimens

All LSFM-dedicated samples were mounted in a 1–2% low melting agarose solution (16520050, ThermoFisher) in PBS by directly mixing the object with the agarose, then pumping the mixture into sample embedding glass cylinder capillaries, selected based on the object’s size and volume (inner diameter of capillary: size 1/∼0.68 mm, size 2/∼1 mm, size 3/∼1.5 mm, size 4/∼2.15 mm, Zeiss). After positioning the specimens using a camera with LED illumination, three-dimensional images were acquired on a LightSheet Z.1 (Zeiss). The system was equipped with multiple solid-state laser lines (405 nm, 445 nm, 488 nm, 515 nm, 561 nm, 638 nm) and two 16-bit sCMOS PCO.Edge cameras for simultaneous dual-color acquisition (1920 x 1920 pixels). The light sheet was focused onto the specimen using Illumination Optics Lightsheet Z.1 5x/0.1 on both the left and right sides. Images were captured with Lightsheet Z.1 detection optics 5x/0.16 NA EC Plan NEOFLUAR 1.33/1.45 adaptive rings, with a z-step of 8–11 μm, a pixel size of 6.5 nm, and a Filter Module LBF 405/488/561/638. Dual-side fusion and relevant processing were performed offline using ZEISS ZEN Black software for Z.1. All images were further post-processed, handled, and automated 3D multiview reconstructed and merged using Fiji software (ImageJ2, version: 2.14.0/1.54f).

#### Generation of iPSC-derived cardiac organoids

Human induced pluripotent stem cells (hiPSCs) specifically used in human cardiac organoids were cultured under feeder-free conditions. Briefly, two hiPSC lines (CMRIi0013-A-6 9CMRI, Stem Cell and Organoid Facility and HPSI0314i-hoik_1 (ECCA)) were maintained on E8 media (Thermofisher, #A1517001) and Geltrex matrix (ThermoFisher, #A1413302) and passaged as clumps with ReleSR (Stem Cell Technologies, #100–0483) upon reaching 60–70% confluence. Cardiac organoids were generated from 70% confluent hiPSCs cultures according to a previously published protocol^78^ with some modifications. Briefly, hiPSCs were dissociated using TrypLE Express (Thermofisher, #12604021) to single cells. Ten thousand cells per 200 μL of E8 (Thermofisher, #A1517001) supplemented with 10 μM Y-27632 ROCK inhibitor (Stem cell Technologies, #72304) were seeded on a low-binding 96-well U-bottom plate (Thermofisher, #174925). The plate was then centrifuged twice (clockwise and anticlockwise) at 200g for 5 min. At day 1 (D1) of differentiation, mesoderm induction was initiated. E8 media was changed to chemically defined media (CDM) (IMDM + GlutaMAX (Thermofisher, #31980030) and Ham F12 + GlutaMAX (Thermofisher, #11765054) in a 1:1 ratio supplemented with 5 mg/mL BSA (Merck, #A1470), 1X Lipid Mix (Merck, #L5146), 1X ITS (Thermofisher, #41400045), 95U/mL PenStrep (Thermofisher, #15070063), 0.4 μg/mL Amphotericin (Thermofisher, #15290018) and 450 μM 1-Monothioglycerol (Merck, #M6145)) supplemented with 4 μM and 6 μM CHIR99201 (Stem cell Technologies, #72054) for HPS10314I-hoik_1 and CMRIi0013-A-6 respectively, 50 ng/mL Activin A (R&D Systems, #338-AC-10), 10 ng/mL BMP4 (R&D Systems, #314-BP-010), 30 ng/mL FGF2 (R&D Systems, #233-FB-025). Three days later (D3), cardiac mesoderm induction started by adding fresh CDM supplemented with 10 ng/mL BMP4 (R&D Systems, #314-BP-010), 8 ng/mL FGF2 (R&D Systems, #233-FB-025), 5 μM IWP-2 (Tocris, #353-310), 0.5 μM Retinoic Acid (Merck, #R2625, 10 μg/mL Insulin (Merck, #I9278), 200 ng/mL VEGF (Peprotech, #AF-100-20). Daily media changes from D3-D7 were performed. On D7, media was changed to CDM supplemented with 10 ng/mL BMP4 (R&D Systems, #314-BP-010), 8 ng/mL FGF2 (R&D Systems, #233-FB-025), 10 μg/mL Insulin (Merck, #I9278) and 100 ng/mL VEGF (Peprotech, #AF-100-20). No media change was required until D10. From D10 onwards, cardiac organoids were maintained on CDM supplemented with 10 μg/mL Insulin (Merck, #I9278) and 100 ng/mL VEGF (Peprotech, #AF-100-20), and feeding was performed every other day. By D12, beating cardiac organoids were transferred to a 6-well plate pre-treated with anti-adherence rinsing solution (Stem Cell Technologies, #07010) and placed on a shaker at 80 RPM. Samples were collected on D12 and D19 for EdU labeling and analysis. hCOs were labeled with 10 μM EdU for 24 h, washed with PBS, and fixed in 4% PFA for 30 min at room temperature. Subsequently, hCOs were stored in 100% methanol at −20°C.

#### Tissue clearing and click labeling of 3D heart organoids

hCOs were rehydrated by incubating in successive dilutions of methanol (75%, 50%, and 25%) prepared in PBSTx (0.3% Triton X-100 in PBS) for 5 min each. This was followed by three washes in PBSTx for 5 min each. Organoids were then permeabilized in ice-cold acetone overnight at −20°C. After permeabilization, organoids were washed three times in PBS for 5 min each. Organoids were then incubated in Solution-1 (25% Urea, 9% THEED, 5% Triton X-100 in water) (DEEP-Clear^49^) overnight (or over the weekend) at 37°C with gentle rocking. To pellet the organoids, tubes were spun briefly in a tabletop microfuge. One milliliter of the supernatant was removed and replaced with PBS. After a few minutes, the organoids became more visible as they turned opaque. Organoids were washed three more times with PBS for 5 min each. Blocking was performed by adding 1 mL of blocking PBSTx (4% FBS, 1% BSA, 1% Triton X-100 in PBS, filtered) to each tube and incubating at 37°C on a rocker for 90–120 min. Most of the supernatant was then removed. OpenEMMU mix was prepared using 1% Triton X-100 in PBS, and 500 μL was added to each tube. Organoids were incubated at 37°C on a rocker for 3 h. Following incubation, tubes were washed three times with PBS and once with 1X Zebrafish perm/wash buffer (0.4% Saponin, 4% FBS, 1% BSA, 10% DMSO, 1% Triton X-100, and 0.02% NaN3) for 10 min each. All but 100 μL of the supernatant was removed. Primary antibodies were prepared in 1X Zebrafish perm/wash buffer, and 100 μL was added to each tube. Tubes were incubated at 37°C on a rocker for 24–48 h (or over the weekend). Tubes were then washed four times with PBSTx (0.3% Triton X-100) for 30–60 min each (or overnight) at 37°C on a rocker, with the first wash lasting 5 min. This was followed by a wash with 1 mL of Zebrafish perm/wash buffer for 10 min at 37°C on a rocker. Most of the supernatant was removed. Secondary and/or fluorochrome-conjugated antibodies (plus Vybrant Violet) were prepared in Zebrafish perm/wash buffer, and 200 μL was added to each tube. Tubes were incubated at 37°C on a rocker for 24–48 h (or over the weekend). Finally, tubes were washed four times with PBSTx for 30 min each at 37°C on a rocker, with the first wash lasting 5 min.

#### *Ex vivo* proliferation of human T cells

##### Antigens

Polyclonal T cell activator anti-CD3/anti-CD28/anti-CD2 was used as a positive control (1/100 dilution; StemCell Technologies, Vancouver, Canada). Recall antigens for memory CD4 T cells included: influenza vaccine (1/100 dilution; Influvac Tetra, 2018 formulation, Mylan Health, Sydney, Australia); *M. avium intracellulare* lysate (5 μg/mL, CSL, Melbourne, Australia); and trimeric recombinant SARS-CoV-2 spike protein (5 μg/mL; which was produced from a plasmid encoding the spike protein with C-terminal trimerization domain and His tag (gift from the Krammer lab, BEI Resources, NIAID, NIH), transfected into Expi93 cells and protein expressed for 3 days, initially purified using the His tag and Talon resin (ThermoFisher), and further purified on a Superose 6 gel filtration column (GE Healthcare) using an AKTA Pure FPLC instrument (GE Healthcare) to isolate the trimeric protein and remove S2 pre-fusion protein, as previously described.^65^^,^^79^

##### CellTrace violet and T-cell proliferation assay

Peripheral blood mononuclear cells (PBMC) were isolated from Na Heparin anti-coagulated blood using Ficoll-Hypaque gradient centrifugation, resuspended at a concentration of 10,000,000 cells/ml in PBS, and incubated with CellTrace™ Violet dye (C34571, Thermofisher) at 5 μM for 20 min at RT, according to the manufacturer’s directions, as previously described.^65^ Cells were washed once with 5x volume of IMDM/10% human serum. Antigen-specific CD4 T cells proliferating in response to recall antigens were measured in cultures of 300,000 PBMC in 200 μL/well of a 96-well plate, in Iscove’s Modified Dulbecco’s Medium (IMDM 12440053; Thermofisher) containing 10% human serum (kind gift, Dr. Wayne Dyer, Australian Red Cross Lifeblood, Sydney, Australia), incubated for 7 days in a 5% CO_2_ incubator.^65^ Different wells contained different antigens as indicated: (i) culture medium only (negative control well); (ii) anti-CD3/anti-CD28/anti-CD2 T cell activator (positive control well); (iii) influenza vaccine (1/100 dilution); and (iv) *M. avium intracellulare* lysate (5 μg/mL); and (v) trimeric recombinant SARS-CoV-2 spike protein (5 μg/mL). After 6 days, cells from the respective cultures were stained with CD3-BV786, CD4-BUV395, CD8-BUV805 and CD25-APC (BD Biosciences), washed once with PBS, fixed in 1% PFA for 30 min at RT and stored at 4°C overnight for OpenEMMU staining with AZDye 488 Picolyl Azide, and acquired on a 5-laser Fortessa X20. Antigen-specific CD4 T cells were gated as CD3^+^CD4^+^CD25^high^CTV^dim^ as previously described.^80^ Cultures were classified as positive for antigen-specific CD4 T cells if the proportion of CD25^high^CTV^dim^ % from CD4^+^ CD3^+^ T cells was ≥1%.^65^^,^^80^

#### EdU labeling of zebrafish embryos and larvae

Screened high-quality 5dpf larvae were incubated with EdU (MedChem Express, HY-118411, 100 mg) at 500 μM concentration in 1% DMSO in Embryo Media for 2 h at 28°C. Following EdU incubation, zebrafish were euthanized in 0.4% Tricaine for 5 min, washed with PBS, and fixed for 1 h at room temperature in either MEMFA (0.1 M MOPS, 2 mM EGTA, 1 mM MgSO_4_, 3.7% PFA, pH 7.4) or 4% PFA, with comparable results obtained from both fixatives. After fixation, samples were washed twice with PBSTx (PBS containing 0.3% Triton X-100), followed by a methanol wash. The methanol was then replaced with fresh methanol, and samples were stored at −20°C overnight or until further processing. Samples can be stored in methanol at −20°C for several months without degradation. For aphidicolin-mediated DNA replication stress experiments, 6 hpf embryos were screened and incubated with 100 μM aphidicolin (MedChemExpress, HY-N6733) in 1% DMSO (v/v) for 20 h at 28°C in E3 medium. At 25 hpf (1 h before the endpoint), embryos were manually dechorionated and incubated in 0.5 mM EdU (1% DMSO) for 1 h at 28°C in E3 medium. Embryos were then fixed in 4% PFA for 30 min and stored as described above.

#### Clearing zebrafish larvae for EdU click-chemistry imaging

5dpf zebrafish larvae were rehydrated by incubating in successive dilutions of methanol (75%, 50%, and 25%) prepared in PBSTx (0.3% Triton X-100 in PBS) for 5 min each. This was followed by two washes in PBSTx for 5 min each. Zebrafish were then permeabilized in ice-cold acetone overnight at −20°C. After permeabilization, zebrafish were washed 4–5 times in PBS. Zebrafish were then de-pigmented in 3% H_2_O_2_ (prepared in 0.8% KOH) for 6–7 min under a bright desk lamp. Following de-pigmentation, zebrafish were washed 4–5 times in PBS. Zebrafish were then incubated in Solution-1.1 (9% THEED, 5% Triton X-100, 5% urea in distilled water) for 10 min at room temperature. After incubation, zebrafish were washed 4–5 times with PBS. Zebrafish were then incubated in zebrafish permeabilization/wash solution (0.4% Saponin, 4% FBS, 10% DMSO, 1% BSA, and 0.02% NaN3 in PBS) for 90 min. Most of the perm/wash buffer was removed, and 400 μL of the OpenEMMU click reaction mix (using 1% Triton X-100 in PBS) was added to each tube. The EdU mix was prepared by sequentially adding the following components: 810 μL PBS, 160 μL of Copper(II) sulfate pentahydrate (209198, Sigma Aldrich; 10 mM fresh or frozen stock, final concentration 800 μM), 3 μL Fluor-Azide (200 μM stock, final concentration 600 nM), and 30 μL freshly made L-Ascorbic acid (100 mg/mL frozen stock, final concentration 17.1 mM). Zebrafish were incubated at room temperature for 60 min. Following click-chemistry incubation, zebrafish were washed once in zebrafish permeabilization/wash solution without DMSO, and most of the supernatant was removed. Vybrant Violet DNA dye was prepared in zebrafish perm/wash solution without DMSO and added to the tubes. Finally, zebrafish were imaged using a suitable microscope. Figures 7D, S11 and 8D images were obtained using a Zeiss Axio Vert.A1 FL-LED inverted microscope with a Microscopy Camera Axiocam 305 color (D) Cooled 5 Mpx 2/3″ CMOS and objective LD A-Plan 5×/0.15 Ph1 M27 (WD = 11.7 mm at D = 1 mm polystyrene).

#### Clearing zebrafish embryos for EdU click-chemistry imaging

PFA-fixed 26hpf zebrafish embryos kept in methanol at −20°C were then permeabilized in ice-cold acetone overnight at −20°C. After permeabilization, zebrafish were washed 4–5 times in PBS. Zebrafish were then de-pigmented in 3% H_2_O_2_ (prepared in 0.8% KOH) for 2 min under a bright desk lamp. Following de-pigmentation, zebrafish were washed 4–5 times in PBS. Zebrafish were then incubated in Solution-1.1 (9% THEED, 5% Triton X-100, 5% urea in distilled water) for 3 min at room temperature. After incubation, zebrafish were washed 4–5 times with PBS. Zebrafish were then incubated in zebrafish permeabilization/wash solution (0.4% Saponin, 4% FBS, 10% DMSO, 1% BSA, and 0.02% NaN3 in PBS) for 60 min. Most of the perm/wash buffer was removed, and 400 μL of the OpenEMMU click reaction mix (using % Triton X-100 in PBS) was added to each tube. The EdU mix was prepared by sequentially adding the following components: 810 μL PBS, 160 μL of Copper(II) sulfate pentahydrate (209198, Sigma Aldrich; 10 mM fresh or frozen stock, final concentration 800 μM), 3 μL Fluor-Azide (200 μM stock, final concentration 600 nM), and 30 μL freshly made L-Ascorbic acid (100 mg/mL frozen stock, final concentration 17.1 mM). Zebrafish were incubated at room temperature for 60 min. Following click-chemistry incubation, zebrafish were washed twice in zebrafish permeabilization/wash solution without DMSO, and most of the supernatant was removed. Alexa Fluor® 594 anti-Histone H3 Phospho (Ser10) antibody (11D8, AB_2801122, BioLegend Cat. No. 650809, 1:200 dilution) and Vybrant Violet™ DNA dye were prepared in zebrafish permeabilization/wash solution (0.4% Saponin, 4% FBS, 10% DMSO, 1% BSA, and 0.02% NaN3 in PBS) and added to the tubes. After over-night incubation, zebrafish were washed 4–5 times with PBS. Zebrafish were z stack imaged using LSM900 confocal microscope.

#### Cardiac resection in adult zebrafish

Cardiac resection and EdU uptake protocols were conducted following previously described methods.^81^^,^^82^ For EdU fluorescent assessment, fixed and frozen hearts were sectioned sagittally at a thickness of 7 μm using a Leica CM1950 clinical cryostat (Leica Biosystems) and collected onto adhesive slides (472042491, Trajan Scientific and Medical). OpenEMMU was then applied to the slides as previously described above for in-house OpenEMMU click chemistry for immunofluorescence and antibody staining, and the nuclei were counterstained with TO-PRO™-3 Iodide (642/661) (T3605, ThermoFisher).

#### Cost analysis of in-house OpenEMMU reagents versus commercial kits

Price calculations were conducted in Australian Dollars (AUD) for each individual off-the-shelf reagent used in the OpenEMMU workflow. These values were subsequently converted to US Dollars (USD) using a fixed exchange rate of 0.64 (as per 28^th^ of May 2025). To standardize comparisons, reaction costs were calculated based on a 0.5 mL reaction volume plus additional steps to ensure completion of the click chemistry OpenEMMU protocol. The total cost of the following click chemistry reagents was included in the analysis: CuSO_4_, Fluoro-Azide, L-Ascorbic Acid, Saponin, Sodium Azide, NCS, PBS, BSA, EdU, DMSO, and PFA. All individual reagent costs were plotted using Prism 10 for macOS (version 10.4.2). These in-house reagent costs were then compared to two commercially available kits: Kit 1 (Click-iT™ Plus EdU Alexa Fluor™ 488 Flow Cytometry Assay Kit, Invitrogen, C10632, 50 assays) and Kit 2 (Click-&-Go Plus 488 Imaging Kit, Vector Labs, VECCT1314), as shown in Figure 1I.

### Quantification and statistical analysis

For statistical analysis, unless otherwise specified, all results obtained from independent experiments are reported as means ± standard errors of means (SEM) of multiple replicates. Unless otherwise indicated, “*n*” in Figure Legends represents the number of animals or independent biological samples or replicates per group.
